# Analysis of a European general wildlife health surveillance program: Chances, challenges and recommendations

**DOI:** 10.1371/journal.pone.0301438

**Published:** 2024-05-21

**Authors:** Elisabeth Heiderich, Saskia Keller, Mirjam Pewsner, Francesco Carlo Origgi, Samoa Zürcher-Giovannini, Stéphanie Borel, Iris Marti, Patrick Scherrer, Simone Roberto Rolando Pisano, Brian Friker, Irene Adrian-Kalchhauser, Marie-Pierre Ryser-Degiorgis

**Affiliations:** 1 Department of Infectious Diseases and Pathobiology, Vetsuisse Faculty, Institute for Fish and Wildlife Health, University of Bern, Bern, Switzerland; 2 Department of Infectious Diseases and Pathobiology, Vetsuisse Faculty, Institute of Animal Pathology (ITPA), University of Bern, Bern, Switzerland; 3 Department of Clinical Research and Veterinary Public Health, Vetsuisse Faculty, Veterinary Public Health Institute, University of Bern, Bern, Switzerland; University of Bucharest, ROMANIA

## Abstract

In a One Health perspective general wildlife health surveillance (GWHS) gains importance worldwide, as pathogen transmission among wildlife, domestic animals and humans raises health, conservation and economic concerns. However, GWHS programs operate in the face of legal, geographical, financial, or administrative challenges. The present study uses a multi-tiered approach to understand the current characteristics, strengths and gaps of a European GWHS that operates in a fragmented legislative and multi-stakeholder environment. The aim is to support the implementation or improvement of other GWHS systems by managers, surveillance experts, and administrations. To assess the current state of wildlife health investigations and trends within the GWHS, we retrospectively analyzed 20 years of wildlife diagnostic data to explore alterations in annual case numbers, diagnosed diseases, and submitter types, conducted an online survey and phone interviews with official field partners (hunting administrators, game wardens and hunters) to assess their case submission criteria as well as their needs for post-mortem investigations, and performed in-house time estimations of post-mortem investigations to conduct a time-per-task analysis. Firstly, we found that infectious disease dynamics, the level of public awareness for specific diseases, research activities and increasing population sizes of in depth-monitored protected species, together with biogeographical and political boundaries all impacted case numbers and can present unexpected challenges to a GWHS. Secondly, we found that even a seemingly comprehensive GWHS can feature pronounced information gaps, with underrepresentation of common or easily recognizable diseases, blind spots in non-hunted species and only a fraction of discovered carcasses being submitted. Thirdly, we found that substantial amounts of wildlife health data may be available at local hunting administrations or disease specialist centers, but outside the reach of the GWHS and its processes. In conclusion, we recommend that fragmented and federalist GWHS programs like the one addressed require a central, consistent and accessible collection of wildlife health data. Also, considering the growing role of citizen observers in environmental research, we recommend using online reporting systems to harness decentrally available information and fill wildlife health information gaps.

## Introduction

**Wildlife health surveillance (WHS) is an integral aspect of national One Health management strategies [1, 2].** The repeated emergence of human and livestock diseases from wildlife origins and the increasing risk of pathogen transmission from wild animals to humans and domestic animals associated with patterns of global change, underline the importance of WHS [3–5]. Emerging diseases in wildlife also contribute to biodiversity loss [6]. For these reasons, WHS constitutes an essential component of national preparedness strategies [1, 7–9]. Accordingly, wildlife health has become a priority for the World Organization for Animal Health (WOAH, formerly OIE) particularly in the wake of the COVID-19 pandemic [10].

**General wildlife health surveillance (GWHS) is a major component of WHS programs**. In veterinary medicine, health has been defined as “a state of physical and psychological well-being and of productivity, including reproduction” [11]. The WOAH defines surveillances as the on-going recording of diseases in animal populations with a view to disease management [12]. The term GWHS stands for a passive system of opportunistic carcass collection that encompasses a wide range of species and diseases [8, 13]. It is often complemented by separate targeted disease- or species-specific focus programs (active or targeted surveillance), and with population monitoring data, to assess epidemiological dynamics, freedom of disease and intervention outcomes in an integrated wildlife monitoring (IWM) program [13].

**The aim of a national GWHS program should be the identification and effective communication of risks to or from the country’s wildlife populations for management [2].** For example, GWHS programs need to assure early detection of emerging pathogens, which is a prerequisite for rapid reactions towards disease control [14–16]. They should also generate appropriate knowledge to improve the effectiveness of wildlife policies and systems [2]. As wildlife diseases do not stop at country borders, harmonization and centralization of information at an international level is of high importance to increase efficiency of national programs [13].

**In practice, however, GWHS faces several challenges [1, 16, 17].** These include aspects related to sample availability, but also to expertise, legal frameworks, and resources. Firstly, GWHS depends on spontaneous reports on wildlife morbidity and mortality by field partners (state game wardens, hunters, wildlife biologists) and from the public. In practice, this means that dead or diseased wildlife must be found, reported, sent for pathological examination, in the shortest possible time to prevent that carcass decomposition may mask the actual pathological lesions, for effective surveillance. Therefore, GWHS can be biased against elusive, common, or non-hunted species. Secondly, GWHS deals with species uncommon to most veterinarians, and often face a lack of validated diagnostic tests, baseline data, harmonized procedures, population data, and expertise [16, 17]. Finally, the benefit of a wildlife health surveillance program to society is difficult to express in metrics, and the fact that wildlife is a public good limits the resources available to GWHS [1, 2, 18].

**GWHS often operates in the face of non-negotiable conditions**. These include (a) the ability of the surveillance system to react to natural developments (e.g., disease outbreaks or changes in population density), (b) the sensitivity of the surveillance system to societal developments (e.g., new laws, mandates, increased attention on biodiversity or One Health), (c) the dependencies and from submitters and funding stakeholders, their needs, limitations, and expertise, (d) the de-facto entanglement and co-dependencies among general surveillance, targeted programs, and research, (e) historically grown and legislatively set boundaries and practices, and (f) potential information gaps associated with all of the above. GWHS is expected to operate and provide valuable insights despite these limitations.

**Fig 1:**
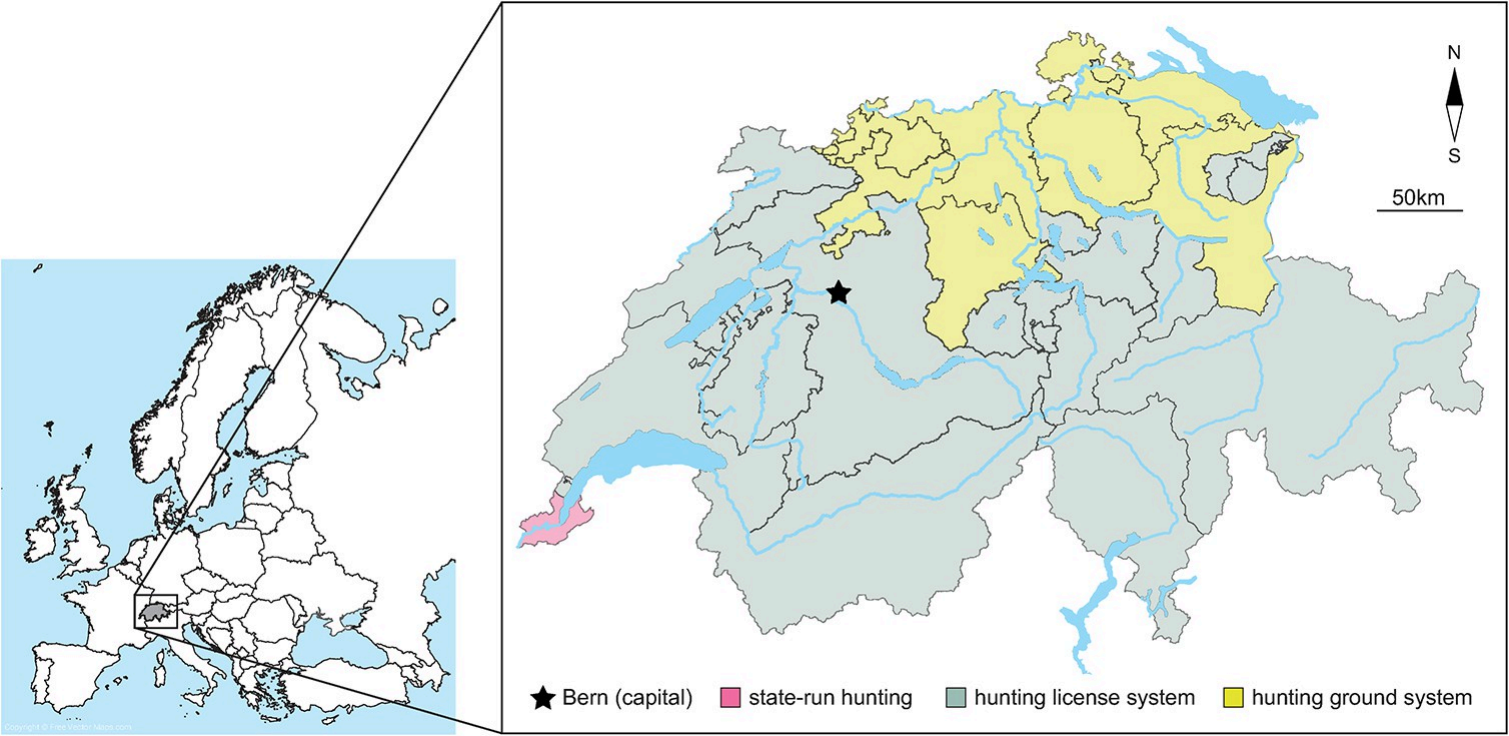
Study setting Switzerland is a federative state comprising 26 states (“cantons”) which feature three distinct hunting regimes. The diagnostic institute is located in the capital city Bern. Map data ©swisstopo.

**The GWHS program analyzed here has a comprehensive scope** of free-ranging terrestrial wildlife, with a focus on mammals, birds, and increasingly reptiles and amphibians. It was started around 1950 in Switzerland [7, 8]. Switzerland is a small federal state (41’285 km^2^), but highly heterogeneous in terms of landscape, climate, habitat, human population density, language, political organization and wildlife management. Twenty-six variably sized, biogeographically distinct, and largely independent political subunits (“cantons”, Fig 1) are further subdivided in wildlife management districts of different sizes [19, 20]. The adjacent Principality of Liechtenstein (160 km^2^) is, for the purpose of veterinary services, affiliated with the Swiss systems.

**The Swiss terrestrial wildlife management system is legally, geographically and administratively fragmented.** The Swiss legal system defines wildlife as *res nullius*, which means they are not the subject of private property but governmentally managed. It is important to understand that wildlife health is regulated by three different laws: the Federal Act on Hunting and the Protection of Wild Mammals and Birds (hunting law) [21], the Federal Act on Animal Diseases (animal disease law) [22] and the Federal Act on the Protection of Nature and Cultural Heritage (nature protection law) [23]. The cantons are responsible for the legal execution of these laws. Each canton features one of three hunting regimes: (1) the hunting license system (16 cantons and the Principality of Liechtenstein), (2) the hunting ground system (nine cantons), and (3) state-run hunting (one canton) (Fig 1). Officially appointed professional state game wardens are present in all cantons with the license system and in the state-run hunting system [24]. In cantons with hunting grounds, these tasks are partly performed by voluntary game wardens. The health surveillance of species not covered in the hunting law is subject to the nature protection law, which is primarily concerned with biodiversity and species protection and conservation and coordinated by the Federal Office for the Environment (FOEN; different division than hunting). The animal disease law focuses on zoonotic diseases or pathogens with high risk of spill-over to domestic and farmed animals, and to any species these pathogens may pertain, and is coordinated and enforced on the federal level by the Federal Food Safety and Veterinary Office (FSVO).

**The Swiss GWHS relies on a single diagnostic institute.** Currently, GWHS for terrestrial animals is conducted at the University of Bern, Veterinary Faculty, Institute for Fish and Wildlife Health (FIWI), hereafter referred to as diagnostic institute, on a mandate of the FOEN. Additional institutions execute targeted surveillance programs for specific pathogens. The diagnostic institute submits carcasses or samples to these programs on occasion for the targeted surveillance of reportable diseases, to prove freedom of reportable diseases or based on pathological signs and suspicion (bats and foxes for rabies, lynx and wolves for trichinellosis, brown hare for tularemia, etc.).

**The GWHS is based on three mandates.** The GWHS covers (a) common free-ranging, middle sized to large wild mammals and birds including those which can be legally hunted (species covered by the hunting law) [21]; (b) eulipotyphla, bats, small rodents, reptiles and amphibians that are not hunted (species covered by the nature protection law). In addition, the diagnostic institute carries out an in-depth health monitoring mandate for protected wild mammals, including the Eurasian lynx (*Lynx lynx*), gray wolf (*Canis lupus*), golden jackal (*Canis aureus*), brown bear (*Ursus arctos*) as well as, until 2019, the Eurasian beaver (*Castor fiber*). The diagnostic institute additionally advices the federal offices, cantonal hunting authorities, game wardens and the public on wildlife disease issues. It provides quarterly reports to the cantonal hunting authorities containing the number of carcasses submitted per species and the diseases/causes of deaths diagnosed.

**The GWHS has characteristics of an academic setting.** The location at the University of Bern comes with certain hallmarks of academic institutions: a dependency on multiple intra- and extra-mural funding sources; the expectation to use submitted cases for teaching; the involvement of fast turn-over trainees and the need for supervision in diagnostics; the availability of in-depth expertise on various diseases and pathogen groups from in-house specialized research groups; the ability, but also the expectation to turn unexpected findings into research projects; the expectation of long-term employees to be able to participate in activities other than diagnostic (own research, teaching, board activities).

**The GWHS operates in a field of partly contrasting needs of the main funding bodies.** Salaries of diagnostics employees are largely funded by the two federal offices, FOEN and FSVO. The university contributes infrastructure and administration, the salary of the institute head and two to three research trainee positions. Submitters are usually not charged for wildlife postmortem examination including further ancillary testing, except for toxicological investigations and postmortem examinations of farmed deer.

The FOEN focuses on population level aspects and insists that general wildlife health surveillance offered at the diagnostic institute meets the legal requirements and the needs of the involved partners concerning pathological investigations, information on current disease dynamics, and education. Largely, the focus of the FOEN relies on species protected by the hunting law. The hunting administrations focus on wildlife management, expect advice in management actions and request case-based support. The FSVO and cantonal veterinary services, in contrast, focus on early warning for emerging diseases, reportable animal diseases and zoonoses, and are thus interested in individual-level data. They do not have a species focus. Finally, the University of Bern (where the diagnostic institute is integrated) focuses on research and teaching tasks. Accordingly, the diagnostic institute needs to maintain, in parallel, a high quality of wildlife health surveillance and diagnostics, teaching activities (requirement by the university for veterinary student education and by the authorities for field partner training), and continuous education of the diagnostic institute’s staff (expertise maintenance).

**This paper provides an unredacted insight into a European GWHS operating within real-world constraints.**

Increased numbers of case submissions for postmortem investigation despite constant funding over the past twenty years motivated this study.

We assessed the following critical points using different methods: (1) A retrospective analysis of the diagnostic data from 2002–19, to identify potential causes for the increased case load; (2) Telephone interviews of program partners, to clarify the needs concerning wildlife health investigations; (3) Online questionnaires for hunting authorities to understand current decision criteria for case selection in the field and to identify potential for improvement; (4) In-house time recordings of post-mortem investigations, to document current time requirements per case and assess the potential for more time efficient workflows. Aim of these analyses was to identify unmet needs and data gaps, suggest measures to make better use of the limited financial resources, and identify future opportunities for GWHS.

## Material and methods

### Retrospective case analysis

**To assess the current state of wildlife investigations and trends within the GWHS**, we analyzed the wildlife diagnostic database of the diagnostic institute between 2002 and 2019. This analysis spans two decades during which diagnostic procedures have remained largely unchanged [25, 26]. Since 2002, wildlife health assessments have been carried out by rotating diagnostic trainees supervised by a board-certified pathologist. The largest change within the study period was the progressive implementation of health assessments in amphibians and reptiles since 2010. The grouping of the investigated species is shown in Table 1.

**Table 1:** Grouping of cases applied during analysis.

Group	Examples of frequent species	Law (mandate)
Protected rodents	Beaver (*Castor fiber*), European squirrel (*Sciurus vulgaris*)	Hunting law
Hunted rodents	Alpine marmot (*Marmota marmota*)	Hunting law
Lagomorphs	European brown hare (*Lepus europaeus*), mountain hare (*L*. *timidus*)	Hunting law
Hunted indigenous carnivores	Red fox (*Vulpes vulpes*), Eurasian badger (*Meles meles*), martens (*Martes* spp.)	Hunting law
Protected indigenous carnivores	Eurasian lynx (*Lynx lynx*), grey wolf (*Canis lupus*), golden jackals (*Canis aureus*)	Hunting law
Non-indigenous carnivores	Raccoon (*Procyon lotor*) and raccoon dog (*Nyctereutes procyonoides*)	Hunting law
Ungulates	Roe deer (*Capreolus capreolus*), Alpine chamois (*Rupicapra rupicapra*), Alpine ibex (*Capra ibex*), wild boar (*Sus scrofa*)	Hunting law
Birds of prey	Buzzards, owls, eagles, falcons, vultures	Hunting law
Land birds	Songbirds (e.g. tits, blackbirds, crows), pigeons, gallliformes	Hunting law
Water birds	Ducks, swans, gulls, herons	Hunting law
Farmed deer	Fallow deer (*Dama dama*), red deer (*Cervus elaphus*) and sika deer (*Cervus nippon*)	-
Domestic animals (diagnostic of predation)	Sheep (*Ovis aries*), goats (*Capra aegagrus*), calves (*Bos taurus*)	-
Eulipotyphla	European hedgehog (*Erinaceus europaeus*)	Nature protection law
Bats	*Myotis* spp., *Pipistrellus* spp.	Nature protection law
Small rodents	Mice (*Muridae*)	Nature protection law
Reptiles	*Natrix* spp.	Nature protection law
Amphibians	Frogs *(Rana* spp.), toads (*Bufo* spp.), salamanders (*Salamandra* spp.), newts (*Triturus* spp.)	Nature protection law

We summarized the number of submitted cases per animal group and diagnosis. Additionally, we described and visualized the distribution of submitted cases by submitter types and the geographical origin during the defined period. The aim of this analysis was to identify fluctuations or trends in numbers of diagnosed causes of death/diseases, submitter types and geographical origin. Maps depicting the origin of the cases were generated with the free software QGIS 3.20.2 [27] and the free geodata from the Office of Topography swisstopo (available from www.swisstopo.ch).

**For comparison purposes, we additionally analyzed the Swiss hunting statistic database between 2011 and 2019.** Cantons are free to decide how, and how much details, they record wildlife found sick, injured or dead, and use a variety of systems from paper trail to online reporting system. However, all cantons feed a minimum amount of information regarding carcass finds into a federal system. Carcasses found dead or with clear signs of disease are filled in the category "age, disease, weakness" (available from www.jagdstatistik.ch). With the numbers pertaining to (1) hunted ungulates, (2) hunted carnivores and (3) lagomorphs (hares) we calculated what percentage of carcasses recorded in the Swiss hunting statistic database was submitted to the GWHS.

### Online survey on case selection in the field

We conducted an online survey (LimeSurvey GmbH, Hamburg, version 4.3.15) with official field partners (hunting administrators, game wardens and hunters) to understand the submitters’ needs and practices regarding submission, health assessment, and information. We contacted the 26 Swiss cantonal hunting administrators and the corresponding person in the Principality of Liechtenstein, as well as the Swiss National Park by email and asked them to (a) fill a form themselves and to (b) forward the survey link to game wardens, park rangers or hunters that were part of their administration. The questionnaire consisted of a maximum of 46 open-ended and close-ended questions, which varied in number and content depending on given answers (S1 Appendix). The first 15 questions collected demographic details of the participants, including name, canton, district of surveillance, function, starting date of the employment in this position, professional education, attended training courses related to wildlife health, and authority for case selection. Answers were a prerequisite to proceed further. Subsequent questions focused on personal criteria of past case submissions, including numbers and incentives, with details on species and further circumstances, such as numbers of affected animals or field observation. We also asked participants to rate the current support concerning case selection (by the cantonal administration and/or the diagnostic institute), whether additional support was deemed necessary and if yes, who should provide it. The online questionnaire was open from July 3rd to August 15^th^ 2020.

For descriptive analysis and statistical analysis, data were exported from the LimeSurvey software to a Microsoft Excel spreadsheet (2016 version, Redmond, Washington, USA). For descriptive statistics, median, mean, minimum and maximum were calculated for continuous variables, and percentages for categorical variables.

Explanatory variables were hunting system, distance from the diagnostic institute (distance between the offices of the cantonal hunting authorities and the center in km as indicated by www.maps.google.com), years of experience, function and level of training of the respondent. Response variables included carcass submission for carcasses with and without visible signs of disease, the frequency of annual submissions of different species groups, and the threshold number of animals after which carcasses were submitted if that many animals were found either without visible signs of disease, with visible signs of a known disease or with visible signs of an apparently new disease, respectively.

The frequencies were recorded as ordinal factor variables with the levels 0, 1–5, 6–10, 11–20 and more than 20. As most respondents chose either one of the highest or one of the lowest option, it was decided to investigate differences between those 2 groups of “low” and “high” submitters, respectively. Thus, the response variables were dichotomized. The threshold was the answer option with the lowest number of responses. This could differ for different species due to their abundance.

For each outcome variable, univariable logistic regression models were run to assess associations with each of the explanatory variables. Variables with p-values <0.2 were taken as the initial variables in the multivariable model for each response variable. Further variable selection was undertaken applying a backward elimination procedure based on the Akaike Information Criterion using the “stepAIC” function of the “MASS” package [29]. The level of statistical significance was set to p = 0.05.

All statistical tests described above were conducted using the R software [28].

### Interviews with national GWHS program partners

To understand the situation and needs of national partners and stakeholders regarding wildlife health assessments, we conducted phone interviews in summer 2020 and fall 2021. Representatives of 1) the 26 cantonal hunting administrations (including the Principality of Liechtenstein) as well as 2) ten non-cantonal institutions (seven wildlife rehabilitation centers; two reptile and two amphibian experts from the Swiss Amphibian and Reptile Conservation Program; one representative from the Swiss National Park) were interviewed between August 17th and September 6^th^ 2020 and between September 8th and October 4th 2021, respectively. Questions were provided ahead of time (S3 and S4 Appendices). The aim was to assess the general satisfaction with the diagnostic institute’s services, and with the current information and communication situation regarding wildlife disease occurrence on the national and international level. Furthermore, we gathered perspectives regarding the interest in, and practical feasibility of, a potential national online reporting system for wildlife diseases. The non-cantonal institutions were additionally asked for their criteria to submit a case for postmortem investigation.

### Ethical approval

This study was reviewed by the Cantonal Ethics Committee for Human Research, Bern, Switzerland (Req-2020-00416) on 10 April 2020 with the result that an ethical approval is not required because it does not fall under the Human Research Act (Art. 2, 1). Written information about the use of their answers for this study was provided to survey participants.

### Assessment of diagnostic effort

To understand caseload-related aspects of diagnostic processes and procedures at the diagnostic institute, a time-per-task analysis was conducted. Between June 2020 and May 2021, four rotating diagnostic trainees with varying levels of experience in wildlife pathology documented the time required to complete certain aspects of postmortem examinations. Trainees recorded pre-/postprocessing activities, necropsy, histology, and reporting (Table 2). The time required to perform these tasks was averaged and recorded for three types of cases (Table 3): protected species cases with in-depth health monitoring, routine cases that included histology, and routine cases without histology. Importantly, the time required for tasks not performed by trainees, e.g., the preparation and staining of histology, the performance of ancillary test (e.g., bacteriology, PCRs) the printing and postage of reports or the archiving of samples and the supervision of the trainees (in the necropsy hall, reading histology slides with trainees and correction of necropsy reports) was not recorded.

**Table 2:** Activities/duties of trainees.

Activity	Examples
Preparation/follow-up	Oral communication with submitter, opening a new case file in the diagnostic software, preparing sampling tubes, processing samples, disposal of material
Necropsy	Including collecting measurements and samples, taking photographs, discussion of case with supervisor, cleaning
Histology	Tissue trimming, slide reading, discussion of slides with supervisor
Reporting	Writing of report draft and corrections required by the involved supervisors

**Table 3:** Types of cases.

Case	Explanation
Protected species (e.g., lynx, wolf, etc.) with in depth-health monitoring	Postmortem examination following standardized procedure with additional tasks including radiology, photographs, morphometrics, weighing organs, systematic sampling for archiving, and histology of main organs
Routine cases of the GWHS with histology	Cases where the cause of death/disease cannot be defined macroscopically and/or by other ancillary tests
Routine cases of the GWHS without histology	Cases where the cause of death/disease can be defined macroscopically and/or by other ancillary tests

## Results

### Case numbers

#### During the 18-year study period, wildlife diagnostic cases investigated at the national diagnostic institute almost tripled

**Fig 2:**
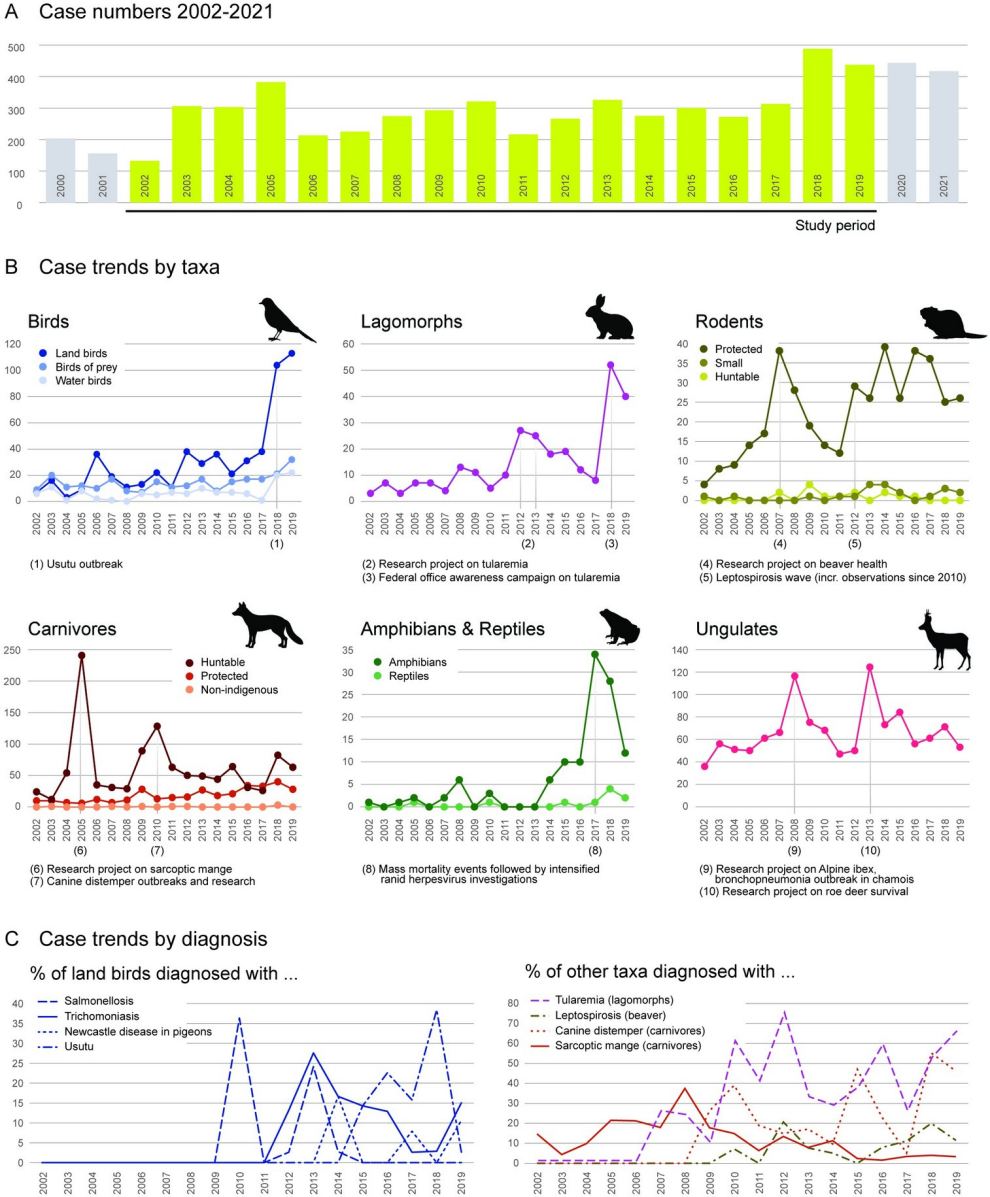
Case submissions. A) Total case numbers. Annual number of carcasses submitted for post-mortem investigation to the diagnostic institute during the study period (2002–2019). B) species-specific submissions. of birds, lagomorphs, rodents, carnivores, amphibians and reptiles (see Table 1 for species grouping) during the study period. C) Pathogen-specific trends. Most common diagnosed diseases in land birds and other taxa/species (lagomorphs, beaver, carnivores) during the study period.

Annual numbers counted a minimum of 132 submissions in 2002, then fluctuated between 200 and 300, except for 2005 with 380 cases per year, until 2017 and reached a maximum of 488 submissions in 2018. The rate of annual case submissions since 2019 until 2021 seems stable at around 400 (Fig 2A).

#### The increased submissions were mainly based on some specific species groups

Marked increases in submissions were observed for birds, lagomorphs, protected rodents, protected carnivores and amphibians. In 2018 and 2019, songbirds constituted about 22% (104/488) and 26% (113/437) of all cases compared to 5% (7/132) in 2002. Similarly, the number of submitted lagomorphs continuously increased since 2002 with a peak in 2018. Numbers of beaver (protected rodent) submissions increased massively from 2002–2007 (in 2007 beavers constituted17% of all cases, 38/225). After a decrease from 2008–2011, numbers fluctuated in a high state until 2017 and decreased to 6% (26/437) of all cases in 2019. Numbers of submitted protected carnivores have risen constantly. Reptiles and amphibians were a relatively minor component of the submitted animal load until 2017, when an marked increase has been observed and maintained in the subsequent years especially for amphibians. Other taxa, such as ungulates, constitute a substantial portion of submitted cases but did not contribute to the upwards trend (Fig 2B). The more recent increase in case load since 2018 is most likely driven by birds, lagomorphs and protected carnivores.

### Causes of death

#### Disease patterns and diagnosis frequencies

The cause of death in submitted birds was often undetermined (33% of all bird cases, 314/942). Four peaks of case submissions were observed due to avian influenza in 2006, frequent salmonellosis in 2009/2010 and 2013 and Usutu in 2018 (59/104 between 2015–2019). While trichomoniasis reached a peak in 2013 repeated peaks of Newcastle Disease of pigeons were recorded in 2014, 2017 and 2019 (Fig 2C).

The most common cause of death of lagomorphs since 2010 was due to tularemia (109/216 between 2010–2019). In protected carnivores with in-depth health monitoring, trauma was most often observed as cause of death, while in hunted species (mostly red foxes) mainly sarcoptic mange and distemper were diagnosed. Mortality events in foxes due to distemper periodically occurred since 2009. The percentage of foxes with sarcoptic mange peaked in 2008 and showed a declining trend since then (Fig 2C). Most frequent diagnoses in amphibians included traumatic events (19/115; 17%) and infectious agents (Herpesvirus 15%; 17/115). The etiology of more than half of the investigated deaths (57%; 66/115) remained undetermined.

### Submission data

#### The diagnostic institute received the majority of carcasses from game wardens and hunters

**Fig 3:**
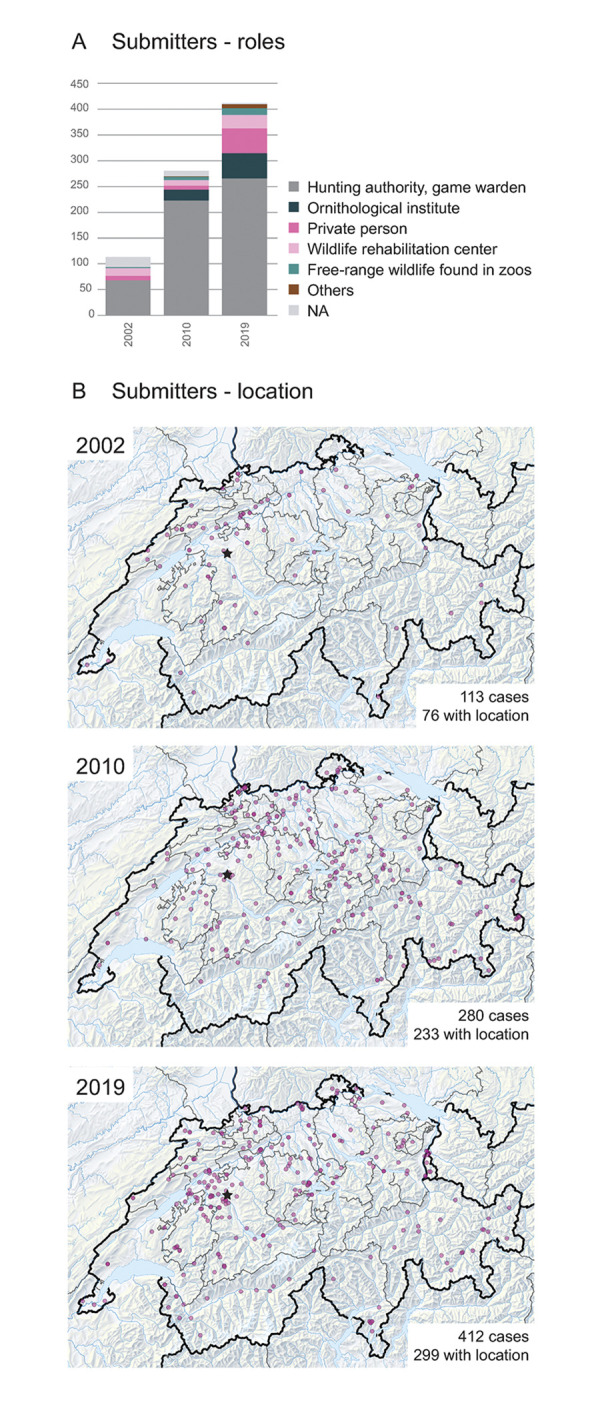
Submission patterns. A) Submitter roles. ~2/3^rd^ of all cases are submitted through hunting-associated stakeholders, while ~1/3^rd^ of cases came from other stakeholders in 2002, 2010, and 2019. The contributions of certain alternative sources are alternating through the years. B) Submitter location. Between 2002 and 2019, the geographic origin of the submissions expanded to previously uncovered regions and administrative units. Map data ©swisstopo.

The retrospective case analysis showed that local authorities (games wardens, hunting administrations, police, veterinary offices) constituted the largest proportion of submitters throughout the study period, with a peak of 80% in 2010 and between 60% and 70% in 2002 and 2019, respectively. Submissions from private persons, wildlife rehabilitation centers and zoos (submission of wildlife found dead/euthanized in the zoo) were subject to larger fluctuations. Contributions from private people have been increasing (3% to 11%) while submissions from the Swiss Ornithological Institute remained relatively constant between 2010 and 2019 (8% and 11%, respectively). Contribution of wildlife rehabilitation centers declined from about 15% in 2002 to 4 and 6% in 2010 and 2019, respectively. Zoos contributed between 2 and 4% of cases throughout the study period (Fig 3A).

#### Biogeographical and political boundaries influenced case submissions

The geographic origin of the submissions was mostly concentrated on the northwestern part of the country in 2002, but the spatial case distribution spread to the eastern and southern parts of Switzerland in 2010 and 2019 (Fig 3B). Statistical analyses of the online survey revealed that the distance to the diagnostic institute was not related to the submissions of carcasses, neither in general nor for those with or without visible disease signs or the number of animals of any species group submitted per year. However the map of the carcass origins showed that carcass submissions from distant cantons were sparse (Fig 3B).

#### Only a small percentage of discovered carcasses or diseased animals were submitted to the GWHS

**Fig 4:**
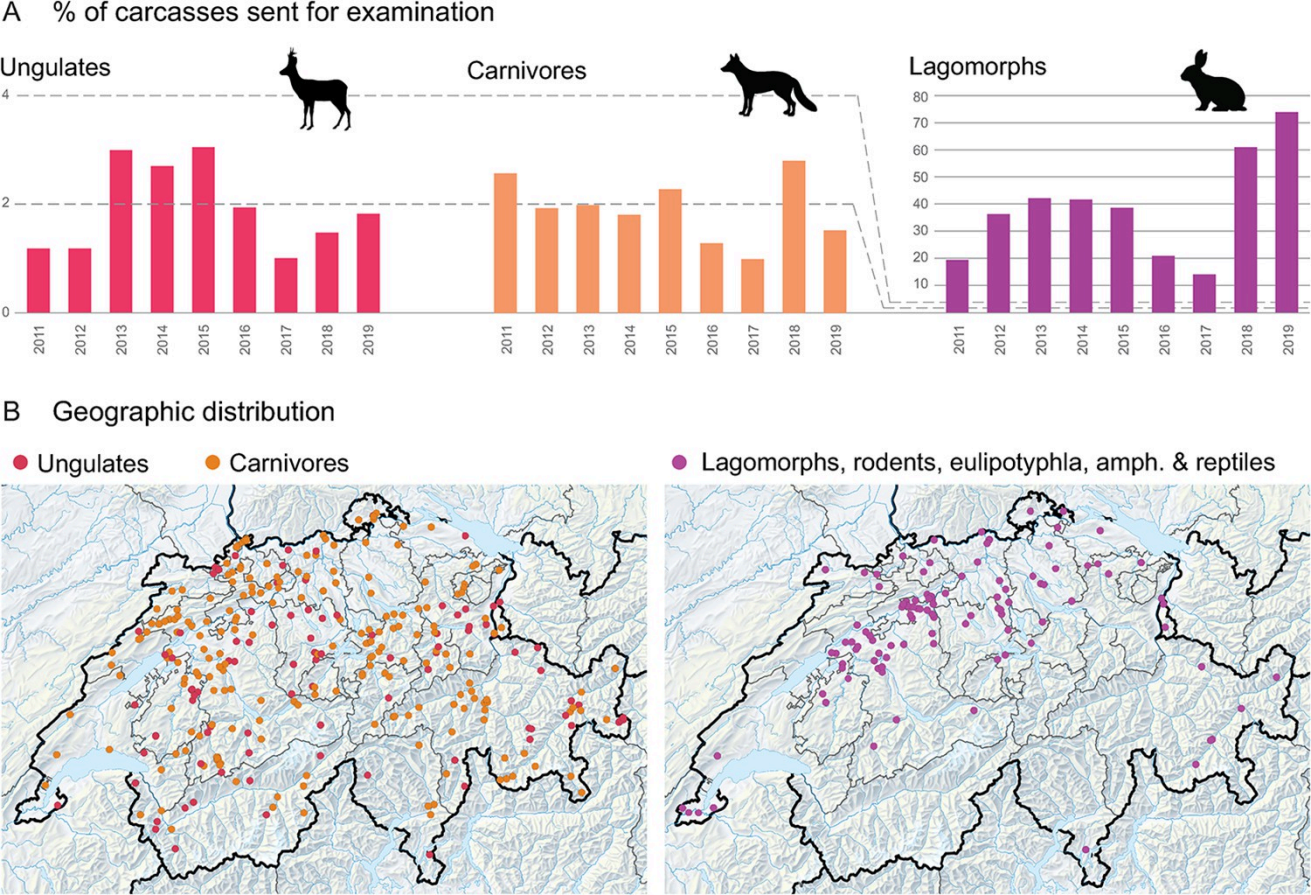
Submission gaps. A) Species gaps. Only ~3% of ungulates and carnivore carcasses were submitted to the GWHS for examination. For lagomorphs the numbers were much higher. B) Geographic gaps. Not all species groups were represented equally in all geographic areas. Data from 2002, 2010 and 2019 together illustrated that ungulates and carnivores (hunted and protected species) were submitted from all over the country, while small species (not hunted) were mostly submitted by areas north of the alps. Map data ©swisstopo.

On average, between one and three percent of animals reported in the Swiss Federal Hunting Statistics in the category "age, disease, weakness" were submitted to the GWHS between 2011 and 2019 (Fig 4A). The proportion of submission was species dependent. For example, between 15% and 75% of lagomorphs found dead were examined at the diagnostic institute with a marked increase since 2018 (Fig 4A). The illustration of the origin of carcasses demonstrated that not all species groups were represented equally in all geographic areas. Data from 2002, 2010 and 2019 together illustrated that ungulates and carnivores (hunted and protected species) were submitted from all over the country, while lagomorphs, rodents, eulipotyphla, amphibians and reptiles (not hunted) were mostly submitted by areas north of the alps. (Fig 4B).

#### The online survey had a high response rate with most of the responders being professional game wardens

We received a total of 238/356 complete questionnaires from 25/26 Swiss cantons as well as the Principality of Liechtenstein, which corresponded to a return rate of 67%. Forty-two percent of the responses were from cantons with a hunting ground system (100/238), 54% from cantons with license system (130/238) and 3% from the canton of Geneva, where hunting is prohibited (8/238). Most participants were professional game wardens (54%, 129/238). The respondents had been in their current position between one month and 42 years (median: 10 years). Most responders had a trade profession as primary education (43%, 102/238). Thirty-eight percent had completed a federal game warden training (90/238) in addition to the cantonal formation. Many others (69%) had taken courses regarding wildlife health (165/238), game meat hygiene (65%, 155/238), wildlife diseases (53%, 127/238). Few had taken courses regarding diagnostic of predation (2%; 4/238) and wildlife immobilization (4%; 1/238) or other courses (6%; 15/238) and 6% have neither completed any courses nor a federal game warden training (15/238). Most respondents (87%; 207/238) were authorized to decide on their own whether a carcass shall be sent for postmortem examination. If not authorized, decision depended on the hunting authorities (81%; 25/31).

#### Cases were submitted to understand the cause of death

Participants indicated they would submit carcasses upon suspicion of an infectious disease (85%; 202/238), to assess the cause of death/disease (62%; 149/238), or upon suspicion of predation (32%; 75/238). Furthermore, half of all responders (55%; 131/238) have already sent dead animals with unknown cause of death and no visible signs of trauma or disease. Carcasses with lesions or clinical signs typical of a specific disease, e.g., sarcoptic mange or distemper, were submitted by only half of all respondents (56%; 134/238), mostly to confirm the field diagnosis (92%; 123/134).

#### Cases were also submitted to satisfy monitoring needs and directives

The online survey participants indicated they would submit carcasses to rule out poaching (22%; 53/238) or hunting outside hunting season (27%; 64/238). A total of 168/238 participants (71%) from 21 cantons indicated that some species were monitored for specific diseases in the framework of targeted health surveillance programs in their canton, and this was also a major reason why carcasses with typical disease signs (see above) were nonetheless submitted to the diagnostic institute or to specialized monitoring programs elsewhere (41%; 55/134). Targeted species and diseases listed by the respondents included wild boar (trichinellosis, African/classical swine fever), brown hares (tularemia, myxomatosis), red deer (tuberculosis, foot rot), waterfowl (avian influenza, Usutu and Newcastle disease), beaver (leptospirosis), fox, martens and badgers (distemper). In line with this, participants most commonly submitted hunted carnivores (63%; 30/48), followed by ruminants (44%; 21/48), protected carnivores (35%; 17/48) and lagomorphs (29%; 14/48).

**Further reasons for submissions** were specific interests of the submitter, e.g., the exclusion of a certain infectious agent (26%; 35/134), and specific requests of the diagnostic institute hosting the GWHS to receive carcasses with suspicion of a specific disease, e.g., for teaching purposes or research projects (18%; 24/134).

#### Cases were not submitted when the infectious cause of death was obvious, or if death was caused by trauma

Responders did not submit carcasses with visible signs of disease if a) they trusted that their field diagnosis was correct (65%; 66/102), e.g., sarcoptic mange, distemper, infectious keratoconjunctivitis; if b) they had received instruction from their authorities, e.g., fox with suspicion of mange, (13%; 13/102) or c) if there were no more requests from laboratories/institutions (3%; 3/102). Trauma cases are also usually not submitted in species with no in-depth health monitoring. A total of 174 out of 238 participants (73%) stated that they have not submitted any carcass with clear evidence of a trauma (e.g., traffic accident) so far. Seven percent (16/238) answered that they send 1–2 animals with signs of trauma per year and 7% (16/238) said they send 5–10 such animals per year. Five percent (12/238) stated 3–4 animals per year and 2% (4/238) send more than ten per year.

#### Case selection in the field encompassed a limited extent of epidemiological considerations

The majority of the responders stated that in case of increased mortality within the same month and district without suspicion of a specific cause of death they would immediately submit a carcass for investigation only if the concerned species was wild boar (36%; 87/238), raccoon or raccoon dog (29%; 70/238) or a protected carnivore (65%; 157/238). For other species groups, 18% (70/238) answered that a minimum of 2–3 carcasses was required prior to first submission. If increased numbers of dead animals with lesions or signs indicative of a suspected disease which is already known within the area were observed, the same answers were given as for animals without signs of disease. Only if protected rodents (beaver) were involved 23% of the participants (54/238) would send the first carcass found for examination. For the same scenario, except with the suspected disease being new to the area, the majority of responders would send the first observed carcass for further investigation, except for hunted carnivores, waterfowl and eulipotyphla. In those cases 31% (73/238), 23% (55/238) and 19% (46/238) would wait until 2–3 dead animals were found within a month, respectively.

#### Hunting systems also affected case selection

For example, wild boar were sent significantly more often from the canton of Geneva (state hunting system) than from cantons with license system (p = 0.02, OR = 1.67, CI = 1.21–2.29). Regarding protected carnivores, respondents from cantons with hunting ground system sent significantly less animals per year than those from cantons with license system (p = 0.04, OR = 0.85, CI = 0.72–0.99).

#### The availability of local veterinary expertise also impacted submission numbers

The majority of respondents who had already submitted carcasses to an institution/laboratory for postmortem examination (77%; 184/238) submitted to the diagnostic institute (70%; 167/238). The other cases were sent to other cantonal or academic laboratories. If responders had not submitted any carcass for necropsy yet, reasons were mostly because they had not had any case of interest (77%; 36/47), they could discuss cases with their local veterinarian (15%; 7/47) or the next laboratory/institute was too far away (4%; 2/47). Almost half of responders indicated that they did contact the cantonal veterinary office before carcass submission (47%; 111/238). Consultation of the cantonal veterinary office prior to carcass submission was done only in case of a suspected reportable disease by 62% (69/111), while 25% (28/111) said that they always do it. Of the responders who do not contact the veterinary office prior to carcass submission, 9% (11/122) contact the office after case submission and 38% (45/122) report to the veterinary office only if a notifiable disease is diagnosed.

#### Subjectively perceived submission numbers were rated to be consistent over the years by most participants

The majority of responders (77%; 184/238) answered they had submitted similar numbers of animals annually in their current function, while 8% (20/238) thought that the number had increased and 8% (19/238) that it had decreased. Six percent (15/238) did not answer the question. The increase was justified in 65% (13/20) as secondary to higher awareness for diseases (e.g., African swine fever, tularemia, avian influenza and tuberculosis), 55% (11/20) to growing populations of protected species (e.g., lynx, beaver, wolf) and 45% (9/20) to the perceived increase of dead or diseased animals (e.g., beaver and orphaned lynx). Main reasons for decrease included a decline of the observed morbidity and mortality (53%; 10/19), (e.g., sarcoptic mange, distemper, rabies), or changed hunting administration instructions (42%; 8/19). Furthermore, the longer a responder had been in service, the more likely they had the impression of decreasing submission numbers (p < 0.001), while there was no relation between the years in service of the responder and the rating of case submissions as increasing (p = 0.56). The complete list of statistical data can be found in the (S5 and S6 Appendices).

#### Interviews and online survey revealed different opinions on the implementation of a nation-wide online reporting system

Phone interviews with the cantonal authorities revealed that fourteen cantons (52%) already work with an online reporting system (ORS) that allows registering wildlife disease occurrence (at various levels of detail). Five additional cantons are planning to set up a digital/app-based system for wildlife management which includes disease recording in the near future. The remaining eight cantons do not have any kind of ORS or do not record wildlife diseases at all. In line with this trend, the majority of hunting authorities (85%; 23/27) and non-cantonal institutions interviewed (100%; 10/10) considered the option to register animals found dead/diseased, but not submitted for pathological investigation useful. Many indicated that such a system would give a better overview of the occurrence and spread of wildlife diseases in Switzerland, and that it could be a useful tool also in connection with existing hunting statistics. While 53% (126/238) of respondents of the online survey did not see an added benefit in a nation-wide ORS, 43% (103/238) expressed views in favor of such a system. Concerns included (1) fear of redundancy and increased efforts. Several electronic systems to record dead wildlife are already in place, and stakeholders were wary of redundant efforts to record cases into a second system. A system that increases administrative effort without yielding substantial benefit at the local level was seen as problematic. Also, concerns focus on (2) reliability. Respondents were concerned that the records would be of questionable informative value as the diseases would only be diagnosed in the field by non-veterinarians and based on external gross lesions. This might bear a risk of misdiagnoses due to the omission of a pathological examination by an expert. Finally, concerns related to (3) the technological requirements of an ORS. Given that ORS require the ability to use an app on a smartphone, respondents pointed towards these requirements as a potential challenge for older hunters.

### Stakeholder’s needs

#### The diagnostic needs of local administration and submitters were currently mostly met

Overall, hunting authorities in Switzerland and Liechtenstein as well as the non-cantonal institutions expressed satisfaction with the services offered by the diagnostic institute concerning wildlife health surveillance. They submit the majority of wildlife carcasses where they a) want to know the cause of death or b) have the suspicion of an infectious disease but cannot determine the disease or c) suspect intoxication. A service which could be expanded, according to stakeholders, is toxicological investigations (i.e., specific laboratory analysis in cases of suspected poisonings), and amphibian health surveillance.

#### Support needs of submitters were also mostly met, and open needs mostly concern cantonal authorities

According to the online survey support from cantonal authorities for decision on case submission was rated as "very good" (55%; 132/238), "good" (28%; 66/238) or "sufficient" (11%; 27/238). Only 1% (3/238) answered "bad" and only one respondent rated it as "very bad" (1/238). The majority (76%; 182/238) stated that they did not need any additional support in this matter from any institution. Thirteen per cent (30/238) named the cantonal veterinary authorities as institution they would like to have more support from, 12% (28/238) from the cantonal hunting administration, 8% (20/238) from the diagnostic institute, 4% (10/238) from the FOEN, 2% (4/238) mentioned the FSVO. One person each mentioned a private veterinarian and the cantonal laboratory.

#### The GWHS did not satisfy non-cantonal stakeholder’s information needs

While the needs of cantonal authorities regarding wildlife disease investigation and information were covered and also benefited from regular inter-cantonal and even international interactions, non-cantonal institutions were less satisfied with the availability of information about wildlife diseases on cantonal and national level. Information requirements were related to biogeography (desire for an online platform or map) and to information access on the local, federal and international level. For example, 8 of 10 non-cantonal institutions stated that they did not have sufficient information about the cantonal and national wildlife disease situation, and four did not have enough information about disease developments and epidemiological trends in Europe.

### Assessment of diagnostic effort

#### Time-tracking of various tasks associated with diagnostics uncovered that postmortem investigations of protected species were the most time-consuming

Postmortem analyses of protected species took on average six hours (median = 334 min, sd = 102 min), which was more than twice as long as necropsies for routine cases (median = 127 min, sd = 80 min). Routine cases with histology took about three hours (median = 164 min, sd = 72 min) compared to one hour for cases without histology (median = 67 min, sd = 82 min). Tasks that do not require veterinary training (e.g., case entry into the system, specimen processing, necropsy preparation, cleaning of necropsy hall) took approximately 17% of the time a veterinarian needed to complete a case (23/139 min).

## Discussion

With this study we wanted to support the implementation or improvement of other real-world GWHS systems by managers, surveillance experts, and administration. We evaluated submission practices and case submission trends in the framework of a European GWHS, with the aim to better understand (1) the impact of disease trends, (2) current case selection practices, (3) overlapping and contrasting needs of stakeholders, and (4) factors impacting the ability of the GWHS to function efficiently and effectively. It uncovered gaps and supports the formulation of recommendations on how to set up or improve wildlife health surveillance programs that face real life challenges and settings.

### Methodological considerations

#### The present study used a multi-tiered approach to understand the current characteristics, strengths and gaps of a small-country European GWHS

We combined a 20-year case analysis, semi-quantitative structured interviews (local administration), an online survey (field experts), and in-house tracking of time-per-case. Response rates in interviews and surveys were excellent, and together with the clear trends in the answers, and the consistent results between independent approaches, the conclusions drawn can be considered valid. A general limitation of the data analysis is the lack of consideration for detection probability of carcasses, which varies depending on species, season, and between partners. This would be beyond the scope of this paper, but certainly affects case submissions and should be kept in mind when discussing trends.Regarding the retrospective case analysis, the observed trends were only based on data visualization. An important point to mention here is the need to consider the effect of taxonomic updates (e.g. for pathogens). During the data analysis procedure, one such update was noticed (Pigeon paramyxovirus 1 vs. Avian paramyxovirus 1).

The overall interpretation of the statistical analysis conducted in this study is limited due to the following reasons: Some factor variable categories had lower sample numbers than others. For example, one hunting system is characteristic for one single canton and thus, there were only few responses. Similarly, some collinearity was detected that was limited to certain factor levels. For example, game wardens had on average more years of experience than people with no function, but there were no more differences between any of the other group-wise comparisons. Nonetheless, having a complete overview of all of Switzerland was deemed more important than statistical accuracy of specific estimates and therefore, it was decided not to omit certain regions of the country or certain groups of experts.

. Another limitation of the assessment of the diagnostic effort is that the individual experience of each trainee, which influences the time-per-case, was not taken into account.

### Case numbers and causes of death

In summary, we found that the observed increased caseload could not be attributed to any singular cause, but was associated with infectious disease dynamics, increased public awareness for specific diseases, research activities and increasing population size of protected species.

#### Disease related factors included the emergence of novel pathogens as well as increased prevalence of known diseases

Songbirds have been increasingly submitted likely in relation to increased disease awareness due to an avian influenza epidemic in 2006 as well as mortality events associated with infectious diseases such as salmonellosis (2010–2013) [28] or the emergence of Usutu (2018) [29] in Europe. In hunted carnivores, case increase was mainly due to infectious disease spread, such as sarcoptic mange [30] and distemper [31, 32]. Distemper simultaneously emerged in surrounding countries and spread up to Denmark [33].

#### Societal factors were related to public perception as well as education

The most submitted species, such as roe deer, red deer, chamois and ibex, are appreciated game species and benefit from increased attention by hunters and hunting authorities. Also, roe deer and foxes are widely distributed throughout the country [34], and often appear close to human settlements. This increases the likelihood of carcasses to be found, which is a typical challenge in wildlife health surveillance [16, 35], but also bears a risk of pathogen transmission to livestock, domestic animals or even humans [36–38]. Generally, however, field experts base their case selection on valid criteria, such as the species, suspected cause of death (infectious vs non-infectious) and apparent local disease emergence.

#### Research projects on focus species, inspired by perceived changes in prevalence or by stakeholder’s needs, sometimes had long-term impacts on submission practices

Research projects contributed to increased submissions of **lagomorphs** (especially European brown hares) for a study on tularemia (2012–13) [39]. Compared to a former study on hare diseases [40], tularemia occurrence significantly increased within 13 years and high case numbers have continued to be recorded since then. This was shown by the high percentages of lagomorph submissions compared to the total number of diseased animals recorded in the Swiss hunting statistics and the marked increase of hare submissions since 2018. High numbers of mangy **foxes** were related to a research project in 2003–05 and to targeted surveillance activities over nearly the whole study period [30, 41]. The increase in **amphibian** submissions since 2017 was due to the setting up of a herpetology network and amphibian health surveillance program, including investigations of mass mortality events [42, 43]. Another reason for increased case submissions were new partnerships, for example with the Swiss Ornithological Institute since salmonellosis detection in passerine birds in 2010, and with the Swiss Amphibian and Reptile Conservation Program since 2013.

#### Finally, increasing population sizes can present unexpected challenges to a GWHS

Increased case submissions of **protected species** were likely most often driven by population growth of Eurasian lynx, grey wolf and beaver [44–46]. For protected carnivores, such as the lynx, wolf and brown bear, Swiss management plans require a full postmortem investigation including non-health related indicators. Similarly, submission of wild cats is strongly encouraged [8]. The increase of protected species burdens the system while not contributing to the GWHS, as every individual needs to get a full postmortem examination, including histopathology and sample storage, even if it was for example a legal culling of a healthy animal. Beavers had an intermediary status, with the same procedure as for wild cats, but enhanced because of a research project in 2006 [47, 48]. Also, **population regulation** can impact the GWHS. For example, wild boar were sent significantly more often from the canton with state hunting than from cantons with hunting permit system, which is likely due to the fact that the canton of Geneva used to have one of the highest wild boar densities [49] and local game wardens carry out population regulation.

#### Together, these results indicate that many small factors can collectively challenge a GWHS, slowly, over time

Short-term increased caseloads due to disease outbreaks can mask long-term gradual change. Accordingly, changes in disease dynamics, in education and awareness, in focus species, and in population sizes need to be accounted for when projecting the capacities and resources of a GWHS. This is particularly relevant if resources are dependent on longer-term cycles (in the presented case, a thorough re-evaluation is possible every four years). It is also relevant to consider development processes, such as slow geographic expansion. For example, the wildlife health surveillance in the form practiced today has been in the process of development over the last 20 years and may not yet represent a steady state system. Therefore, a combination of early-on definition of clear aims regarding the mandates of various stakeholders is recommended, as are regular discussions regarding implementation and execution of these mandates in view of current situations and developments.

### Submission data

#### For GWHS with a strong focus on hunted species, the value of submissions from private persons, wildlife rehabilitation centers and species centers are sometimes questioned by the federal authorities

However, so-called garden wildlife (species such as hedgehogs or songbirds) is almost exclusively submitted by these submitter groups [50], and also not recorded in the national hunting statistics database. Diseases such as Usutu, or salmonellosis, are frequent in garden wildlife, which is usually in close contact with pets, farm animals, and humans. Promoting the submission of these non-hunt-related groups to the GWHS is key to early disease detection and monitoring in the context of a One Health perspective.

#### Another question when improving or building GWHS is whether to rely on a single diagnostic center or multiple dispersed centers

The majority of wildlife carcasses were submitted to the diagnostic institute despite the presence of other institutions that perform veterinary pathological examinations. Additionally, the majority of responders indicated that the veterinary authority was not informed before case submission and many of them did not contact the veterinary authorities at all. At the same time, the Swiss Ordinance on Epizootic Diseases requires since 2017 that hunters and game wardens inform an official veterinarian in case of suspicion of a notifiable disease in a free-ranging wild animal [51]. The role of local veterinarians is therefore mixed, and likely still evolving. On the one hand, cantonal veterinary institutions primarily work with domestic animals and often lack expertise in wildlife health. On the other hand, including local veterinary expertise more in case selection, or in a notification system for systematic data collection, might be advisable to close some gaps of the current GWHS.

### Stakeholder needs

#### Operating in a multi-stakeholder environment is both a challenge and an opportunity for a GWHS

The analyzed system is located at the interface of distinct funding bodies and distinct areas of responsibilities. Somewhat counterintuitively yet typical for multi-stakeholder settings in public good areas, resources to fulfill its role need to be predicted, budgeted, and negotiated by the GWHS itself. The presence of different funding sources leaves the GWHS in the position to negotiate and balance the stakeholder’s needs and ensure that everyone’s needs are met without compromising each other’s resources. Also, external funding comes with reporting obligations that are resource intensive. At the same time, the situation is a win for each stakeholder. None of them could satisfy their legally mandated needs on their own without substantially increasing invested resources, or even potentially competing, e.g., cases for teaching vs diagnostics. To ensure that stakeholder’s needs are met by the GWHS; a decision tree for case submissions has been developed at the diagnostic institute together with the stakeholders involved in the GWHS (S7 Appendix). This decision tree is tailored to the legal framework and goals of the GWHS, the expectations of the mandating national authorities and the university, as well as the needs of the field partners. It is important to note, that such a decision tree needs to be regularly adapted to changing circumstances.

#### While most needs are currently met by the GWHS, systematic, timely and centrally accessible information on the occurrence and prevalence of wildlife diseases is lacking

While clear processes and central information systems exist for livestock diseases, there is no central information record in Switzerland for the occurrence of non-notifiable and common diseases (e.g., distemper, sarcoptic mange). As a result, the information situation on the occurrence of those diseases is perceived as poor, especially by stakeholders of animals rarely examined at the diagnostic institute. Solutions to this information gap are discussed below.

### Demanding objectives

The combination of multiple, sometimes diverging needs also leads to a situation where certain aspects are both a blessing and a burden. Both aspects presented below may impact the diagnostic efficiency. From a scientific perspective, however, they are a treasure trove of otherwise unavailable information. For example, the processing of trauma cases of protected species requires a careful and continuous balancing of extracting the maximum of information when case numbers are low, and reducing efforts when case numbers rise. Here, the ability to immediately react and take decisions together with all stakeholders is key for the functioning of a diagnostic institute.

**In-depth monitored protected species** (wolf, lynx, wildcat, golden jackal, beavers) present a particular challenge for the diagnostic institute. They are time-consuming and take almost three times as long as routine cases. Reasons for this are that postmortem investigations of these species include x-rays, the collection of morphometric data of the carcass and the inner organs, as well as organ sampling for histopathology and biobanking. Often, they also have to be dissected in a carcass-preserving manner for voucher and museum preservation. Additionally, the academic teaching setting with the associated high personnel turnover and the high fraction of trainees with different degrees of experience effects time per case. However, given the lower cost of trainees, longer time per case due to the academic setting does not translate to higher resource needs. Minor reductions on time per case are possible through efficiency-enhancing measures. However, this does not address the main issue for the GWHS: a large proportion of these carcasses do not contribute to the focus of the GWHS, as they often represent healthy individuals killed by trauma. Nevertheless, the apparent prevalence of pathogens found in these species is much closer to the real prevalence compared to any other species group examined. Any reductions in efforts are annihilated by the increasing population sizes of protected species in the area.

**Histopathology** also contributes substantially to time spent per case. However, it has a number of advantages crucial to disease surveillance, including the detailed classification of lesions, the detection of tissue alterations that are not visible macroscopically [52] and the demonstration of associations between pathogen identification and tissue lesions [26, 42]. In addition, in the academic setting of the described GWHS, histopathology–decision, sample preparation, and slide evaluation–is a key part of personnel training, in particular of post-graduate students. Carefully weighing the benefits of histopathological examinations in relation to the relevance of the case for wildlife health surveillance (individual animal vs. population problem), questions of the submitter (animal welfare, zoonosis), informative value of the additional information obtained (fresh condition of the carcass) and added value for teaching is therefore warranted to account for the priorities of all stakeholders and funders.

### Gaps

#### The GWHS features pronounced information gaps which are region and species specific

In line with previous studies [25, 26], there are large, region and species specific, discrepancies between animals found dead or diseased according to the Swiss hunting statistic database and the number of cases investigated in the context of the Swiss GWHS. **Firstly,** the surveillance gap concerns diseases with overt signs such as infectious keratoconjunctivitis in wild ibex and chamois [53], sarcoptic mange in carnivores [30] or foot rot in ungulates [54], and other obvious causes of mortality (e.g., traffic accidents) are typically diagnosed in the field. Submitters specified that cases with obvious cause of death or if death was caused by trauma, were not submitted which supports this statement. However, field diagnoses yield the risk of missing underlying conditions, e.g., a fox with trauma as final cause of death might have been weakened by an infectious disease, such as distemper, which made him prone to be hit by a car. Cantons are at liberty to record these cases as they see fit and report them to the national hunting statistics database as "age, disease, weakness" or "traffic", without further indication of disease etiology. This leads to the situation that wildlife health experts face a lack of accessible comprehensive overview of wildlife disease occurrence on the national level, even though the data is actually recorded to some extent at the local level and causes an information gap within the GWHS with regard to certain, often frequent, diseases. **Secondly**, the focus of the GWHS on the hunting and game system causes blind spots in species that are not covered by the hunting law, that are not relevant in a hunting context, or that are only occasionally hunted. For example, reptiles, amphibians, bats, eulipotyphla, small rodents and raccoons/raccoon dogs were not named among commonly submitted animal groups by the survey respondents. These species are not covered by the nature protection law wherefore the mandate for disease monitoring is not as clearly formulated. In circumstances that are legally not entirely clear, personal interest, passion and initiative play a significant role. An excellent example for this are amphibians and reptiles. These were originally not covered by the diagnostic institute (given its original focus on hunted species), but personal interest of a new hire led to increasing collaborations and case numbers, which led to federally financed research projects, and finally (in 2020) to an official mandate for amphibian and reptile health monitoring. **Thirdly,** biogeographic aspects and habitat accessibility play a role in submissions. While detection of fresh wildlife carcasses is an intrinsic challenge of any GWHS [12, 29], this is particularly challenging for remote alpine areas where access as well as submission logistics, e.g., cooling and shipment, can be difficult. Therefore, alpine species such as marmots (Marmota marmota) or alpine snow grouses (Lagopus muta) are almost non-existent in the GWHS, even though they are not rare.

#### In addition to the gaps in the data, our analyses reveal three types of issues with regard to data access

**Firstly**, a large amount of data on wildlife health is collected locally. There is no central collection or consolidation of this wealth of information. **Secondly**, specialist disease centers responsible for targeted surveillance schemes currently report to the FSVO in real-time, but this information is only shared by monthly update for reportable diseases and with even bigger delay for other diseases such as tuberculosis or trichinellosis, and only within administration and not between diagnostic centers. This leads to situations where several centers independently start to suspect a disease outbreak. **Thirdly**, currently no wildlife health data is shared systematically between the WHS (targeted or general) and species focus centers, such as the Swiss Ornithological Institute or the Bat Foundation Switzerland. The exchange of health data between centers, a prerequisite for an integrated wildlife monitoring [13], currently relies on personal acquaintance and communication, with the exception of amphibians and reptiles (official case reporting and annual summary of diagnoses since 2017).

### Measures

#### Practice-science collaborations, such as represented by GWHS programs, are capable of flexible incorporation of change

**Fig 5:**
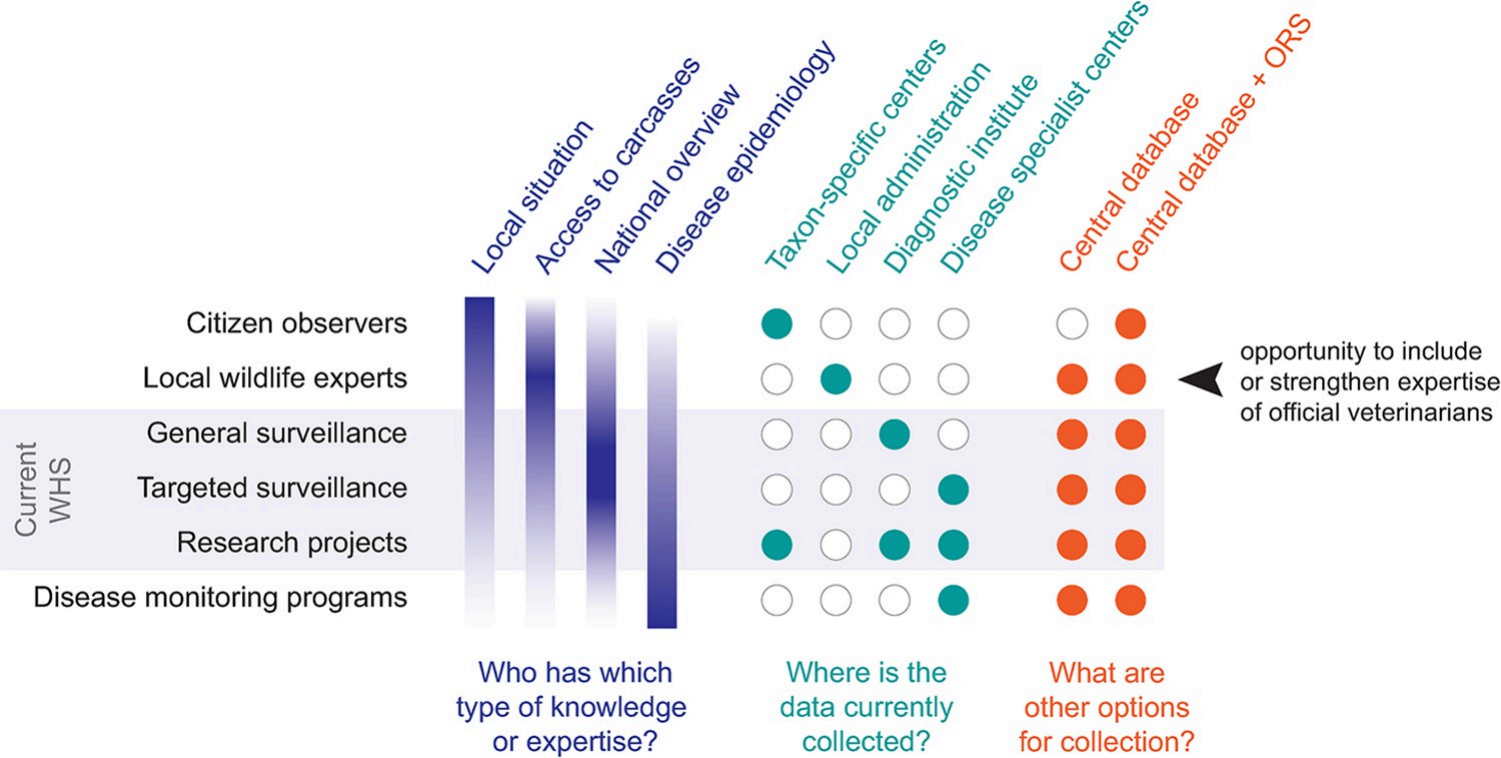
Knowledge and data sources, collection points, gaps, and recommendations. Wildlife health surveillance (WHS) knowledge and expertise is scattered across various levels of geographic areas and expertise. Citizens, local experts, general surveillance, targeted surveillance, research projects, and specialist centers each contribute different types of expertise (violet), and their knowledge is currently collected at different sites (teal). More holistic collection options would include a central database that is accessible for all contributors, and the inclusion of online reporting systems (ORS) in the data collection process (orange). Importantly, local veterinarians are presently not allowed to handle/treat wildlife except for euthanasia to end suffering, and official veterinarians are usually not trained to recognize or deal with wildlife disease.

Fig 5 illustrates how different types of information contributors have distinct types of expertise regarding wildlife health. Integrating method-driven, theory-based, and validated scientific knowledge with real-world, action-based, and contextualized experimental knowledge is a key aspect of sustainable system transitions and system changes [55]. We recommend that people in charge of accompanying such change processes are familiar with basic concepts of transdisciplinarity [56] its application in multidisciplinary and multi-stakeholder policy contexts [57] and factors that increase the success chance of such projects [58]. Incorporating learnings from other fields that navigate the knowledge-to-policy line and that are forced to continuously incorporate past, present, and predicted change such as maritime spatial planning [59] or groundwater policy [60] may be useful. For example, successful policy change processes in the sustainability context tend to (i) engage stakeholders in a participatory process that includes collaborative modeling and social learning; (ii) provide improved understanding of evolving scenarios from all perspectives relevant to the topic; (iii) acknowledge and address uncertainty in scientific knowledge as well as the diversity of stakeholder preferences, e.g. using multi-model uncertainty analysis; (iv) adopt transdisciplinary monitoring and evaluation methods from the very start [60]. Other factors that play a role are (1) the maturity of relationships within the collaboration, (2) the level of context knowledge present within the collaborative team, and (3) the intensity of the engagement efforts within the project [58].

#### A variety of measures could address the apparent information needs and information gaps identified in this study

Some of them are straightforward to implement–others require a concerted effort from a multitude of partners. **Firstly**, current annual and quarterly reports which summarize relevant findings in the submitted cases issued by the diagnostic institute retrospectively at regular intervals are currently available only to a limited set of recipients in the federal offices and cantonal administration. Provided that data protection issues can be solved satisfactorily, these reports could be additionally distributed to rehabilitation centers and other non-cantonal institutions or made freely available on an online repository. **Secondly**, fragmented and federalist systems like the one presented absolutely require a central, consistent and accessible collection of numbers of animals found dead or diseased in a central database (Fig 5). Otherwise, efforts of multiple actors at multiple levels go to waste. Recently, the federal authorities have launched a project to incorporate more health-related data in the hunting statistics. A key part of such a central data collection is to include not just the resources for the database and the information management, but also for regular, real-time, modeling-based, epidemiological evaluation of the data to understand remaining blind spots and opportunities as well as needs for targeted disease surveillance. **Thirdly**, the potential of the GWHS in guiding case selection and in reporting data could be expanded or strengthened (Fig 5). So far, field diagnoses have rarely been reported to the GWHS, resulting in information gaps in national records and therefore in reports to the World Organization of Animal Health and knowledge gaps in general. The surveillance gap in rare or poorly detectable species can be covered by targeted research projects, or “focus species” efforts. An inclusion of local veterinary experts would create additional needs with regard to training and education, a need that the GWHS host institution is able to expertly fill thanks to its academic setting. **Fourthly**, online reporting systems (ORS) represent an option to harness decentrally available information, and thus fill wildlife health information gaps (Fig 5). A few countries have implemented ORS, for example based on mobile technologies [61]. Mobile-phone based reporting systems provide almost real-time data, which proved practicable especially in lower-resource or remote rural settings [62]. A study using an internet-based surveillance system to monitor avian influenza activities showed that such a systems might complement traditional surveillance systems and thus contribute to early detection of disease outbreaks [63]. Therefore, the establishment of an ORS for wildlife diseases and mortality in Switzerland has great potential to improve the knowledge of wildlife health across the country and to acknowledge the value of the collected data. If compatible with wildlife population monitoring systems or linked to other institutions, it could even generate synergies between wildlife disease surveillance and wildlife population monitoring, leading to so-called integrated monitoring [9, 13] with the possibility of generating combined cartographic data. It would be desirable for such an ORS not to be limited to selected diseases, but to allow for reporting clinical signs and visible lesions (the base of the so called “syndromic surveillance”) as well as tentative (field) diagnoses for all animals found dead [64, 65]. According to our results, most hunting authorities would support the introduction of an ORS. However, a project to introduce a nationwide uniform ORS would have to take existing concerns (additional effort, misdiagnosis, technical challenges) seriously and address them proactively and in a sociologically sound manner. At the same time, the information technology challenges (links to different databases, targeted access to certain data categories) would have to be professionally addressed by the IT side. Finally, it would have to be clarified in a legally secure manner where such a database can be located, who undertakes systematic evaluations and who gets access to the data. **Finally**, citizen observers can play a key role in wildlife health surveillance, especially in non-hunted species. The role of citizen science for targeted or general surveillance of wildlife disease including syndromic approaches has been explored previously. However, given the risk of zoonotic diseases, carcass collection and wild animal handling should only be undertaken by professional staff or with appropriate personal protective equipment [50]. Several citizen science reporting systems exist in Switzerland, mostly targeted at reporting native or invasive plants (www.infoflora.ch), bird sightings (www.ornitho.ch), but also general species occurrences (www.infofauna.ch). ORS could potentially incorporate diseases or syndromes, and feed into a central WHS database (Fig 5). ORS based on field diagnoses carry the risk of missed diagnoses, i.e., diseases that are not recognized in the field because they lack clear visible signs and of misdiagnoses, i.e., the visible signs cannot clearly be assigned to one disease (e.g., central nervous symptoms). Therefore, the deployment of an ORS must include a) information of the submitter’s expertise and experience, b) some kind of measure of certainty (either added in the field or post-hoc based on submitter role, and c) an evaluation of how important it is in a given epidemiological setting to get each and every field diagnosis right. For some diseases, an ORS may allow to gather information where there was none at all before, which can be very valuable despite an inevitable certain level of mistakes. A risk-benefit analysis should therefore be part of ORS implementation and evaluation.

## Conclusions and recommendations

For stakeholders and administration facing the challenge of building, improving or adapting a general wildlife health surveillance program, we offer the following recommendations. **Firstly**, changes in disease dynamics, in education and awareness, in focus species, and in population sizes need to be accounted for when projecting the capacities and resources of a GWHS. In particular, increasing population sizes of protected species can present unexpected challenges to a GWHS if their health is monitored in-depth. In a multi-stakeholder scenario, communicable, clear criteria might facilitate case selection and communication of caseload developments. Therefore, we suggest the development of careful case definitions and a decision tree that is tailored to the goals of a general wildlife health surveillance program, to the expectations of the mandating national authorities, and the needs of field partners. We also recommend engaging in regular open exchange to spot and discuss changing settings early on. **Secondly**, we recommend keeping an eye out for information gaps in common or easily recognizable diseases, species that are not hunted, or regions that are most distant to the monitoring institute, and to implement annual or bi-annual focus species programs to cover any systematic gaps through targeted surveillance programs. **Thirdly**, we recommend the systematic implementation of data sharing processes between all institutions involved in aspects of wildlife health monitoring which should be independent from personnel connections. Also, considering the growing role of citizen observers in environmental research, and the data gap for garden wildlife in a hunting-heavy surveillance system, the inclusion of citizen observations seems advisable. For both issues, an ORS represents an option to harness decentrally available information, and thus fill wildlife health information gaps.

## Supporting information

S1 Appendix Survey questions and answers. (PDF)

S2 Appendix Variables. (XLSX)

S3 Appendix Questions needs assessment of hunting administrations. (PDF)

S4 Appendix Questions needs assessment of non-cantonal institutions. (PDF)

S5 Appendix Overview of univariable analysis. (XLSX)

S6 Appendix Overview of multivariable analysis. (XLSX)

S7 Appendix Decision tree. (PDF)

## References

[1] Lawson B, Neimanis A, Lavazza A, López-Olvera JR, Tavernier P, Billinis C, et al. How to start up a national wildlife health surveillance programme. Animals. 2021;11: 2543. doi: 10.3390/ani11092543 34573509 PMC8467383

[2] Stephen C, Duff JP, Gavier-Widen D, Ryser-Degiorgis MP, Uhart MM, Sleeman J, et al. Proposed attributes of national wildlife health programmes. Rev Sci Tech OIE. 2018;37: 925–936. doi: 10.20506/37.3.2896 30964459

[3] Daszak P, Cunningham AA, Hyatt AD. Emerging infectious diseases of wildlife—threats to biodiversity and human health. Science. 2000;287: 443–449. doi: 10.1126/science.287.5452.443 10642539

[4] Bengis RG, Leighton FA, Fischer JR, Artois M, Mörner T, Tate CM. The role of wildlife in emerging and re-emerging zoonoses. Rev Sci Tech. 2004;23: 497–511. doi: 10.20506/rst.23.2.1498 15702716

[5] Gortázar C, Ferroglio E, Höfle U, Frölich K, Vicente J. Diseases shared between wildlife and livestock: a European perspective. Eur J Wildl Res. 2007;53: 241. doi: 10.1007/s10344-007-0098-y

[6] Cunningham AA, Daszak P, Wood JLN. One Health, emerging infectious diseases and wildlife: two decades of progress? Philos Trans R Soc B Biol Sci. 2017;372: 20160167. doi: 10.1098/rstb.2016.0167 28584175 PMC5468692

[7] Kuiken T, Ryser-Degiorgis MP, Gavier-Widen D, Gortazar C. Establishing a European network for wildlife health surveillanc. Rev Sci Tech OIE. 2011;30: 755–761. doi: 10.20506/rst.30.3.2067 22435188

[8] Ryser-Degiorgis M-P, Segner H. National competence center for wildlife diseases in Switzerland: Mandate, development and current strategies. Schweiz Arch Für Tierheilkd. 2015;157: 255–266. doi: 10.17236/sat00019 26753341

[9] Barroso P, Relimpio D, Zearra JA, Cerón JJ, Palencia P, Cardoso B, et al. Using integrated wildlife monitoring to prevent future pandemics through one health approach. One Health. 2023;16: 100479. doi: 10.1016/j.onehlt.2022.100479 36600947 PMC9806683

[10] World Organisation for Animal Health. OIE wildlife health framework—protecting wildlife health to achieve One Health". 2021 [cited 7 Apr 2022]. Available: https://www.woah.org/fileadmin/Home/eng/Internationa_Standard_Setting/docs/pdf/WGWildlife/A_Wildlifehealth_conceptnote.pdf

[11] Studdert VP, Gay CC, Hinchcliff KW. Comprehensive veterinary dictionary. 5th edition. London, United Kingdom: W. B. Saunders; 2020.

[12] Artois M, Bengis R, Delahay RJ, Duchêne M-J, Duff JP, Ferroglio E, et al. Wildlife disease surveillance and monitoring. In: Delahay RJ, Smith GC, Hutchings MR, editors. Management of disease in wild mammals. Tokyo: Springer Japan; 2009. pp. 187–213. doi: 10.1007/978-4-431-77134-0_10

[13] Cardoso B, García-Bocanegra I, Acevedo P, Cáceres G, Alves PC, Gortázar C. Stepping up from wildlife disease surveillance to integrated wildlife monitoring in Europe. Res Vet Sci. 2022;144: 149–156. doi: 10.1016/j.rvsc.2021.11.003 34815105

[14] Kruse H, Kirkemo A-M, Handeland K. Wildlife as source of zoonotic infections. Emerg Infect Dis. 2004;10: 2067–2072. doi: 10.3201/eid1012.040707 15663840 PMC3323390

[15] Mörner T, Obendorf DL, Artois M, Woodford MH. Surveillance and monitoring of wildlife diseases. Rev Sci Tech OIE. 2002;21: 67–76. doi: 10.20506/rst.21.1.1321 11974631

[16] Ryser-Degiorgis M-P. Wildlife health investigations: needs, challenges and recommendations. BMC Vet Res. 2013;9: 223. doi: 10.1186/1746-6148-9-223 24188616 PMC4228302

[17] Sonnenburg J, Ryser-Degiorgis M-P, Kuiken T, Ferroglio E, Ulrich RG, Conraths FJ, et al. Harmonizing methods for wildlife abundance estimation and pathogen detection in Europe—a questionnaire survey on three selected host-pathogen combinations. BMC Vet Res. 2016;13: 53. doi: 10.1186/s12917-016-0935-x 28202055 PMC5312528

[18] Kock RA. Is it time to reflect, not on the “what” but the “why” in emerging wildlife disease research? J Wildl Dis. 2019;55: 1–2. doi: 10.7589/2019-01-000 30412006

[19] Burri K. Schweiz Suisse Svizzera Svizra. Geographische Betrachtungen ab 7. Schuljahr. Schülerbuch. Lehrmittelverlag; 1998.

[20] Schneider-Sliwa R. Schweiz: Geographie, Geschichte, Wirtschaft, Politik. Darmstadt: WBG (Wissenschaftliche Buchgesellschaft); 2011.

[21] Federal act on hunting and the protection of wild mammals and birds. JSG, 922.0 Apr 1, 1988. Available from: https://www.fedlex.admin.ch/eli/cc/1988/506_506_506/de

[22] Federal act on animal diseases. TSG, 916.40 Jul 1, 1966. Available from: https://www.fedlex.admin.ch/eli/cc/1966/1565_1621_1604/de

[23] Federal act on the protection of nature and cultural heritage. NHG, SR, 451 Jul 1, 1966. Available from: https://www.fedlex.admin.ch/eli/cc/1966/1637_1694_1679/de

[24] Imesch-Bebié N, Gander H, Schnidrig-Petrig R. Ungulates and their management in Switzerland. European ungulates and their management in the 21st century. New York: Cambridge University Press; 2010. pp. 392–418.

[25] Akdesir E, Origgi FC, Wimmershoff J, Frey J, Frey CF, Ryser-Degiorgis M-P. Causes of mortality and morbidity in free-ranging mustelids in Switzerland: necropsy data from over 50 years of general health surveillance. BMC Vet Res. 2018;14: 195. doi: 10.1186/s12917-018-1494-0 29921290 PMC6009050

[26] Pewsner M, Origgi FC, Frey J, Ryser-Degiorgis M-P. Assessing fifty years of general health surveillance of roe deer in Switzerland: a retrospective analysis of necropsy reports. PLoS ONE. 2017;12: e0170338. doi: 10.1371/journal.pone.0170338 28103325 PMC5245894

[27] QGIS.org Geographic information system. Boston, Massachusetts, USA: Open Source Geospatial Foundation Project; 2020. Available: https://qgis.org

[28] Giovannini S, Pewsner M, Hüssy D, Hächler H, Degiorgis M-PR, Hirschheydt J von, et al. Epidemic of salmonellosis in passerine birds in Switzerland with spillover to domestic cats. Vet Pathol. 2013;50: 597–606. doi: 10.1177/0300985812465328 23125146

[29] Clé M, Beck C, Salinas S, Lecollinet S, Gutierrez S, Van de Perre P, et al. Usutu virus: a new threat? Epidemiol Infect. 2019;147: 1–11. doi: 10.1017/S0950268819001213 31364580 PMC6625183

[30] Pisano SRR, Zimmermann F, Rossi L, Capt S, Akdesir E, Bürki R, et al. Spatiotemporal spread of sarcoptic mange in the red fox (Vulpes vulpes) in Switzerland over more than 60 years: lessons learnt from comparative analysis of multiple surveillance tools. Parasit Vectors. 2019;12: 521. doi: 10.1186/s13071-019-3762-7 31690337 PMC6833187

[31] Origgi FC, Plattet P, Sattler U, Robert N, Casaubon J, Mavrot F, et al. Emergence of canine distemper virus strains with modified molecular signature and enhanced neuronal tropism leading to high mortality in wild carnivores. Vet Pathol. 2012;49: 913–929. doi: 10.1177/0300985812436743 22362965

[32] Sattler U, Khosravi M, Avila M, Pilo P, Langedijk JP, Ader-Ebert N, et al. Identification of amino acid substitutions with compensational effects in the attachment protein of canine distemper virus. J Virol. 2014;88: 8057–8064. doi: 10.1128/JVI.00454-14 24807725 PMC4097785

[33] Yon L, Duff JP, Ågren EO, Erdélyi K, Ferroglio E, Godfroid J, et al. Recent changes in infectious diseases in European wildlife. J Wildl Dis. 2019;55: 3–43. doi: 10.7589/2017-07-172 30284963

[34] Schweizerische Gesellschaft für Wildtierbiologie, Graf R, Fischer C. Atlas der Säugetiere—Schweiz und Liechtenstein. 1. Auflage. Bern: Haupt Verlag; 2021.

[35] Stallknecht DE. Impediments to wildlife disease surveillance, research, and diagnostics. Berlin Heidelberg: Springer Verlag; 2007. 10.1007/978-3-540-70962-6_17 17848074

[36] Pisano SRR, Ryser-Degiorgis M-P, Rossi L, Peano A, Keckeis K, Roosje P. Sarcoptic mange of fox origin in multiple farm animals and scabies in humans, Switzerland, 2018. Emerg Infect Dis. 2019;25. doi: 10.3201/eid2506.181891 31107228 PMC6537710

[37] Schöning JM, Cerny N, Prohaska S, Wittenbrink MM, Smith NH, Bloemberg G, et al. Surveillance of bovine tuberculosis and risk estimation of a future reservoir formation in wildlife in Switzerland and Liechtenstein. PLoS ONE. 2013;8: e54253. doi: 10.1371/journal.pone.0054253 23349839 PMC3549981

[38] Wu N, Abril C, Thomann A, Grosclaude E, Doherr MG, Boujon P, et al. Risk factors for contacts between wild boar and outdoor pigs in Switzerland and investigations on potential Brucella suis spill-over. BMC Vet Res. 2012;8: 116. doi: 10.1186/1746-6148-8-116 22817843 PMC3464720

[39] Origgi FC, Frey J, Pilo P. Characterisation of a new group of Francisella tularensis subsp. holarctica in Switzerland with altered antimicrobial susceptibilities, 1996 to 2013. Eurosurveillance. 2014;19. doi: 10.2807/1560-7917.es2014.19.29.20858 25080140

[40] Haerer G, Nicolet J, Bacciarini L, Gottstein B, Giacometti M. Todesursachen, Zoonosen und Reproduktion bei Feldhasen in der Schweiz [Causes of death, zoonoses, and reproduction in the European brown hare in Switzerland]. Schweiz Arch Tierheilkd. 2001;143: 193–201. doi: doi.org/10.5169/seals-591650 11344944

[41] Nimmervoll H, Hoby S, Robert N, Lommano E, Welle M, Ryser-Degiorgis M-P. Pathology of sarcoptic mange in red foxes, (Vulpes vulpes): macroscopic and histologic characterization of three disease stages. J Wildl Dis. 2013;49: 91–102. doi: 10.7589/2010-11-316 23307375

[42] Origgi FC, Otten P, Lohmann P, Sattler U, Wahli T, Lavazza A, et al. Herpesvirus-associated proliferative skin disease in frogs and toads: proposed pathogenesis. Vet Pathol. 2021;58: 713–729. doi: 10.1177/03009858211006385 33813961

[43] Origgi FC, Schmidt BR, Lohmann P, Otten P, Akdesir E, Gaschen V, et al. Ranid herpesvirus 3 and proliferative dermatitis in free-ranging wild common frogs (Rana temporaria). Vet Pathol. 2017;54: 686–694. doi: 10.1177/0300985817705176 28494706

[44] Carnivore Ecology and Wildlife Management (KORA). Available from: https://www.kora.ch/en/. Accessed 23 Jun 2023.

[45] info fauna Nationales Daten- und Informationszentrum der Schweiz. Available from: https://infofauna.ch/de. Accessed 23 Jun 2023.

[46] Chapron G, Kaczensky P, Linnell JDC, von Arx M, Huber D, Andrén H, et al. Recovery of large carnivores in Europe’s modern human-dominated landscapes. Science. 2014;346: 1517–1519. doi: 10.1126/science.1257553 25525247

[47] Marreros N, Zürcher-Giovannini S, Origgi FC, Djelouadji Z, Wimmershoff J, Pewsner M, et al. Fatal leptospirosis in free-ranging Eurasian beavers (Castor fiber L.), Switzerland. Transbound Emerg Dis. 2018;65: 1297–1306. doi: 10.1111/tbed.12879 29673086

[48] Wimmershoff J, Robert N, Mavrot. Causes of mortality and diseases in the reintroduced European beaver populations in Switzerland from 1989 to 2009. Proceedings of the Joint EWDA/WDA Conference. Lyon, France; 2012. p. 37.

[49] Meier R, Ryser-Degiorgis M. Wild boar and infectious diseases: evaluation of the current risk to human and domestic animal health in Switzerland: a review. Schweiz Arch Für Tierheilkd. 2018;160: 443–460. doi: 10.17236/sat00168 29989552

[50] Lawson B, Petrovan SO, Cunningham AA. Citizen science and wildlife disease surveillance. EcoHealth. 2015;12: 693–702. doi: 10.1007/s10393-015-1054-z 26318592

[51] Swiss ordinance on animal diseases. TSV, 916.401 Sept 1, 1995. Available from: https://www.fedlex.admin.ch/eli/cc/1995/3716_3716_3716/de

[52] Ryser-Degiorgis M-P, Robert N, Meier RK, Zürcher-Giovannini S, Pewsner M, Ryser A, et al. Cardiomyopathy associated with coronary arteriosclerosis in free-ranging Eurasian lynx (Lynx lynx carpathicus). Front Vet Sci. 2020;7: 594952. doi: 10.3389/fvets.2020.594952 33409296 PMC7779598

[53] Mavrot F, Vilei EM, Marreros N, Signer C, Frey J, Ryser-Degiorgis M-P. Occurence, quantification, and genotyping of Mycoplasma conjuctivae in wild caprinae with and without infectious keratoconjunctivitis. J Wildl Dis. 2012;48: 619–631. doi: 10.7589/0090-3558-48.3.619 22740528

[54] Moore-Jones G, Dürr S, Willisch C, Ryser-Degiorgis M-P. Occurence of footrot in free-ranging Alpine ibex (Capra ibex) colonies in Switzerland. J Wildl Dis. 2021;57: 327–337. doi: 10.7589/JWD-D-20-00050 33822150

[55] Scholz RW, Steiner G. Process ownership in science–practice collaborations: the special role of transdisciplinary processes in sustainable transitioning. Sustain Sci. 2023;18: 1501–1518. doi: 10.1007/s11625-023-01291-7

[56] Lawrence MG, Williams S, Nanz P, Renn O. Characteristics, potentials, and challenges of transdisciplinary research. One Earth. 2022;5: 44–61. doi: 10.1016/j.oneear.2021.12.010

[57] Basta C, Kunseler E, Wamsler C, van der Jagt A, Baró F, Balenciaga I, et al. Inclusiveness, equity, consistency, and flexibility as guiding criteria for enabling transdisciplinary collaboration: lessons from a European project on nature-based solutions and urban innovation. Front Clim. 2021;3: 630075. doi: 10.3389/fclim.2021.630075

[58] Ferguson DB, Meadow AM, Huntington HP. Making a difference: planning for engaged participation in environmental research. Environ Manage. 2022;69: 227–243. doi: 10.1007/s00267-021-01585-5 34999911 PMC8789721

[59] Gissi E, Fraschetti S, Micheli F. Incorporating change in marine spatial planning: a review. Environ Sci Policy. 2019;92: 191–200. doi: 10.1016/j.envsci.2018.12.002

[60] Elshall AS, Arik AD, El-Kadi AI, Pierce S, Ye M, Burnett KM, et al. Groundwater sustainability: a review of the interactions between science and policy. Environ Res Lett. 2020;15: 093004. doi: 10.1088/1748-9326/ab8e8c

[61] Mwabukusi M, Karimuribo ED, Rweyemamu MM, Beda E. Mobile technologies for disease surveillance in humans and animals. Onderstepoort J Vet Res. 2014;81: 5 pages. doi: 10.4102/ojvr.v81i2.737 25005126

[62] Robertson C, Sawford K, Daniel SLA, Nelson TA, Stephen C. Mobile phone–based infectious disease surveillance system, Sri Lanka. Emerg Infect Dis. 2010;16: 1524–1531. doi: 10.3201/eid1610.100249 20875276 PMC3294391

[63] Yousefinaghani S, Dara R, Poljak Z, Bernardo TM, Sharif S. The assessment of Twitter’s potential for outbreak detection: avian influenza case study. Sci Rep. 2019;9: 18147. doi: 10.1038/s41598-019-54388-4 31796768 PMC6890696

[64] Kelly TR, Pandit PS, Carion N, Dombrowski DF, Rogers KH, McMillin SC, et al. Early detection of wildlife morbidity and mortality through an event-based surveillance system. Proc R Soc B Biol Sci. 2021;288: 20210974. doi: 10.1098/rspb.2021.0974 34256001 PMC8277475

[65] Warns-Petit E, Morignat E, Artois M, Calavas D. Unsupervised clustering of wildlife necropsy data for syndromic surveillance. BMC Vet Res. 2010;6: 56. doi: 10.1186/1746-6148-6-56 21162732 PMC3018415

