# Supplementary material for: Analysis of a European general wildlife health surveillance program: Chances, challenges and recommendations
Source: PLoS One. 2024 May 21;19(5):e0301438. doi: 10.1371/journal.pone.0301438 (PMC11108157; doi:10.1371/journal.pone.0301438)
Supplement: S1 Appendix — (PDF) [file pone.0301438.s001.pdf]

Complete survey results

Part 1: General information [***Teil 1:*** *Allgemeine Angaben*]

1. Surname [*Vorname*]
2. Name [*Name*]
3. Phone number [*Telefonnummer*]
4. Email adress [*Email-Adresse*]
5. Date [*Datum*]
6. Canton (single choice, n=238)
   [*Kanton (Einfachantwort, n=238)*]
   - AG: 4 (1.68%
   - AI: 1 (0.42%)
   - AR: 0
   - BS: 1 (0.42%)
   - BL: 1 (0.42%)
   - BE: 23 (9.66%)
   - FR: 13 (5.46%)
   - GE: 8 (3.36%)
   - GL: 4 (1.68%)
   - GR: 52 (21.85%)
   - JU: 4 (1.68%)
   - LU: 3 (1.26%)
   - NE: 2 (0.84%)
   - NW: 1 (0.42%)
   - OW: 3 (1.26%)
   - SH: 4 (1.68%)
   - SZ: 1 (0.42%)
   - SO: 45 (18.91%)
   - SG: 8 (3.36%)
   - TG: 31 (13.03%)
   - TI: 8 (3.36%)
   - UR: 3 (1.26%)
   - VD: 13 (5.46%)
   - VS: 1 (0.42%)
   - ZG: 1 (0.42%)
   - ZH: 2 (0.84%)
   - FL: 1 (0.42%)

7. In which supervisory area/district/institution do you currently operate? (n=238)
   Designation/number or if no designation: Postal code of municipality(ies)
   [*In welchem Aufsichtsgebiet/Revier/Institution sind Sie im Moment tätig?(n=238)*
   Bezeichnung/Nummer bzw. falls keine Bezeichnung: Postleitzahl der Gemeinde(n)]

8. In what function do you serve in this supervisory area? (multiple choice, n=238)
   Please select the answers that apply:
   [*In welcher Funktion sind Sie in diesem Aufsichtsgebiet tätig?(Mehrfachantwort, n=238)*
   Bitte wählen Sie die zutreffenden Antworten aus:]
   - Game warden [*Wildhüter*]*:* 129 (54.20%)
   - Hunting warden [*Jagdaufseher/in*]*:* 83 (34.87%)
   - Park ranger SNP [*Parkwächer/in SNP*]: 2 (0.84%)
   - Hunter [*Jäger/in*]: 61 (25.63%)
   - Hunting inspector [*Jagdinspektor/in*]: 11 (4.62%)
   - Other [*Sonstiges*]: 36 (15.13%)

9. How long have you been working in this position in the current supervisory area/district/institution? (n=238)
   If you do not know the day and month, please fill in with 01.01.
   [*Seit wann sind Sie in dieser Funktion im jetzigen Aufsichtsgebiet/Revier/Institution tätig?(n=238) Wenn Sie Tag und Monat nicht wissen, bitte mit 01.01. ausfüllen*]

10. What profession did you learn? (n=238)
    [*Welchen Beruf haben Sie erlernt? (n=238*)]
    - Biologist [*Biologe*]: 12 (5.04%)
    - Academic background (medicine related) [*Akademiker mit medizinischen Hintergrund*]: 3 (1.26%)
    - Academic background (not medicine related) [*Akademiker ohne medizinischen Hintergrund*]: 7 (2.94%)
    - Trade profession [*Handwerklicher Beruf*]: 102 (42.86%)
    - Medical/chemical education [*Medizinisch/chemischer Beruf*]: 5 (2.10%)
    - Forestry/agriculture/fishery/environment related [*Forst/Landwirtschaft/Fischzucht/Umwelt*]: 68 (28.57%)
    - Other [*Sonstiges*]: 41 (17.23%)

11. Do you have a federal game warden training? (single answer, n=238)
    Please choose one of the following answers:
    [*Haben Sie eine eidgenössische Wildhüterausbildung?(Einfachantwort n=238) Bitte wählen Sie eine der folgenden Antworten*:]
    - Yes [*Ja*]: 90 (37.82%)
    - No [*Nein*]: 148 (62.18%)

12. In addition to hunting or game warden training, have you taken any courses related to wildlife diseases? (single answer, n=238)
    Please choose one of the following answers:
    [*Haben Sie zusätzlich zur Jagd- bzw. Wildhüterausbildung Kurse in Bezug auf Wildtierkrankeiten besucht?(Einfachantwort, n=238)*
    Bitte wählen Sie eine der folgenden Antworten:]
    - Yes [*Ja*]: 165 (69.33%)
    - No [*Nein*]: 73 (30.67%)

13. Which courses have you attended? (if "yes" to question 12, n=165)
    Please select the answers that apply:
    [*Welche Kurse haben Sie besucht? (wenn "ja" bei Frage 12, n=165) Bitte wählen Sie die zutreffenden Antworten aus*:]
    - Game meat hygiene course [*Wildbrethygienekurs*]: 155 (65.13%)
    - Wildlife diseases [*Krankheiten Wildtiere*]: 127 (53.36%)
    - Other [*Sonstiges*]: 20 (8.40%)

14. Do you have the authority to decide whether an animal should be sent for investigation of the cause of death/illness? (single choice, n=238)
    Please choose one of the following answers:
    [*Sind Sie befugt zu entscheiden, ob ein Tier zur Untersuchung der Todes-/Krankheitsursache eingeschickt wird?(Einfachantwort, n=238)*
    Bitte wählen Sie eine der folgenden Antworten:]
    - Yes [*Ja*]: 207 (86.97%)
    - No [*Nein*]: 31 (13.03%)

15. Who makes the initial decision whether to send an animal for investigation of cause of death/illness? (if "no" to question 14, single choice, n=31)
If you do not have the authority to independently decide whether to send an animal in for investigation of cause of death/illness, please answer the following questions based on when you would contact your supervisor regarding sending it in.
Please choose one of the following answers
[*Wer entscheidet an erster Stelle, ob ein Tier zur Untersuchung der Todes-/Krankheitsursache eingeschickt wird?(wenn "nein" bei Frage 14, Einfachantwort, n=31)*
Falls Sie nicht befugt sind, selbstständig zu entscheiden, ob ein Tier zur Untersuchung der Todes-/Krankheitsursache eingeschickt wird, beantworten Sie bitte die folgenden Fragen, bezogen auf wann Sie Ihren Vorgesetzten bezüglich einer Einsendung kontaktieren würden.
Bitte wählen Sie eine der folgenden Antworten]
    - Hunting authorities [*Jagdverwaltung*]: 25 (80.65%)
    - Cantonal veterinarian [*Kantonstierarzt*]: 2 (6.45%)
    - Others [*Sonstiges*]: 4 (12.90%)

Part 2: Submissions of dead or killed wild animals (or their organs) for examination [***Teil 2****: Einsendungen von toten oder erlegten Wildtieren (oder deren Organe) zur Untersuchung*]

16. Have you ever sent animals or their organs to a laboratory/institute for investigation of cause of death/disease? (single choice, n=238)
Please select one of the following answers:
[*Haben Sie schonmal Tiere oder deren Organe zur Untersuchung der Todes-/Krankheitsursache an ein Labor/Institut geschickt?(Einfachantwort, n=238)*
Bitte wählen Sie eine der folgenden Antworten:]
    - Yes [*Ja*]: 184 (77.31%)
    - No [*Nein*]: 51 (21.43%)
    - No answers [*Keine Antwort*]: 3 (1.26%)

17. To which laboratory/institute did you send the animals or organs? (if " yes" to question 16. multiple choice, n=238)
Please select the answers that apply:
[*An welches Labor/Institut haben Sie die Tiere geschickt?(wenn "ja" bei Frage 16, Mehrfachantwort, n=238)*
Bitte wählen Sie die zutreffenden Antworten aus:]
    - Institute for Fish and Wildlife Health (FIWI), University of Bern [*Institut für Fisch-und Wildtiergesundheit (FIWI), Universität Bern*]: 167 (70.17%)
    - Institute for Veterinary Pathology, University of Zurich [*Institut für Veterinärpathologie, Universität Zürich*]: 23 (9.66%)
    - Institute for Bacteriology, University of Zurich [*Institut für Veterinärbakteriologie, Universität Zürich*]: 7 (2.94%)
    - Institute Galli-Valerio, Lausanne [*Institut Galli-Valerio, Lausanne*]: 32 (13.45%)
    - Cantonal laboratory [*Kantonales Labor*]: 39 (16.39%
    - Other [*Sonstiges*]: 19 (7.98%)

18. Which cantonal laboratory did you send the animals or organs to? (if "cantonal laboratory" in question 17, n=32)
[*Um welches kantonale Labor handelt es sich? (falls "kantonales Labor" bei Frage 17 gewählt wurde, n=32)*]

- Office for Food Safety and Animal Health, Canton Graubünden [*Amt für Lebensmittelsicherheit und Tiergesundheit, Canton of Graubünden*]: 19 (59.38%)
- Biology Laboratory, Chur [*Biologie-Labor Chur*]: 2 (6.25%)
- Cantonal laboratory, Freiburg [*Kantonales Labor, Freiburg*]: 4 (12.50%)
- Cantonal veterinary service, Canton of Solothurn [*Kantonaler Veterinärdienst, SO*]: 2 (6.25%)
- Veterinary diagnostic laboratory, Canton of Sankt Gallen [*Labor für Veterinärdiagnostik, SG*]: 1 (3.13%)
- Others [*Sonstiges*]: 4 (12.50%)

19. For what reason? (if "no" to question 16. multiple choice, n=46)
Please select the answers that apply
[*Aus welchem Grund?(falls "nein" bei Frage 16, Mehrfachantwort, n=46)*
Bitte wählen Sie die zutreffenden Antworten aus:]
    - I did not have a relevant case so far [*Ich hatte bisher keinen entsprechenden Fall*]: 36 (78.26%)
    - I can answer the relevant questions with the local veterinarian on site [*Ich kann die entsprechenden Fragen mit dem lokalen Tierarzt vor Ort beantworten*]: 7 (15.22%)
    - The nearest examination laboratory is too far away (packing, sending or bringing is too inconvenient) [*Das nächste Untersuchungslabor ist zu weit weg (verpacken, schicken bzw. bringen ist zu umständlich)*]: 2 (4.35%)
    - Other [*Sonstiges*]: 2 (4.35%)

20. For what reason do you send whole animals or their organs to a laboratory/institute for examination? (multiple choice, n=238)
Please select the answers that apply:
[*Aus welchem Grund schicken Sie ganze Tiere oder deren Organe zur Untersuchung an ein Labor/Institut? (Mehrfachantwort, n=238)*
Bitte wählen Sie die zutreffenden Antworten aus:]
    - Examination for infectious disease/epidemic [*Untersuchung auf infektiöse Krankheit/Seuche*]: 202 (84.87%)
    - Determination of the cause of death [*Feststellung der Todesursache*]: 149 (62.61%)
    - Health monitoring of protected animal species that must be examined at FIWI according to the Federal Office for the Environment (bear, wolf, lynx, wildcat) [*Gesundheitsüberwachung geschützter Tierarten, die gemäss Bundesamt für Umwelt (BAFU) am FIWI untersucht werden müssen (Bär, Wolf, Luchs, Wildkatze)*]: 89 (37.39%)
    - Diagnostic of predation [*Rissdiagnostik*]: 75 (31.51%)
    - Suspicion of poaching [*Verdacht auf Wilderei*]: 53 (22.27%)
    - Examination of mammary glands (lactation yes/no?) [*Untersuchung von Milchdrüsen (Laktation ja/nein?)*]: 64 (26.89%)
    - Other [*Sonstiges*]: 12 (5.04%)

21. Are there certain animal species (or samples) in your canton that must be sent to a specific laboratory/institute or to the veterinary office for health monitoring with regard to selected diseases/pathogens? (single choice, n=238)
Please select one of the following answers:
[*Gibt es in Ihrem Kanton bestimmte Tierarten (bzw. Proben), die zur Gesundheitsüberwachung im Hinblick auf ausgewählte Krankheiten/Erreger an ein bestimmtes Untersuchungslabor/-institut oder an das Veterinäramt geschickt werden müssen? (Einfachantwort, n=238)*
Bitte wählen Sie eine der folgenden Antworten:]
    - Yes [*Ja*]: 168 (70.59%)
    - No [*Nein*]: 65 (27.31%)
    - No answer [*Keine Antwort*]: 5 (2.10%)

22. Which animal species is/are involved? (if "yes" to question 21, multiple choice, n=168)
If you check the "Other" box, please indicate which animal species is/are involved, what disease is/are being tested for, and what samples is/are being sent for this purpose.
Please select the answers that apply:
[*Um welche Tierart(en) handelt es sich? (falls "ja" bei Frage 21, Mehrfachantwort, n=168)*
Falls Sie das Feld "Sonstiges" ankreuzen, geben Sie bitte an, um welche Tierart(en) es sich handelt, auf welche Krankheit untersucht wird und welche Proben dafür eingesendet werden.
Bitte wählen Sie die zutreffenden Antworten aus:]
    - Wild boar [*Wildschweine*]: 122 (72.62%)
    - Waterfowl [*Wasservögel*]: 36 (21.43%)
    - Deer [*Hirsche*]: 36 (21.43%)
    - Hares [*Hasen*]: 69 (41.07%)
    - Other [*Sonstiges*]: 32 (19.05%)

23. What disease(s) are the wild boar tested for? (if "wild boar" to question 22, multiple choice, n=122)
Please select the answers that apply:
[*Auf welche Krankheit(en) werden die Wildschweine untersucht?(falls "Wildschein" bei Frage 22 ausgewählt wurde, Mehrfachantwort, n=122)*
Bitte wählen Sie die zutreffenden Antworten aus:]
    - Trichinella [*Trichinen*]: 114 (93.44%)
    - African/Classical Swine Fever [*Afrikanische/Klassische Schweinepest*]: 83 (68.03%)
    - Other [*Sonstiges*]: 8 (6.56%)

24. What disease(s) are the waterfowl tested for? (if "waterfowl" to question 22., multiple choice, n=36)
Please select the answers that apply:
[*Auf welche Krankheit(en) werden die Wasservögel untersucht? (falls "Wasservögel" bei Frage 22 ausgewählt wurde, Mehrfachantwort, n=36)*
Bitte wählen Sie die zutreffenden Antworten aus:]
    - Avian influenza [*Vogelgrippe/Aviäre Influenza*]: 34 (94.44%)
    - Other [*Sonstiges*]: 2 (5.56%)

25. What disease(s) are the deer tested for? (if "deer" to question 22., multiple choice, n=34)
Please select the answers that apply:
[*Auf welche Krankheit(en) werden die Hirsche untersucht? (falls "Hirsche" bei Frage 22 ausgewählt wurde, Mehrfachantwort, n=34)*
Bitte wählen Sie die zutreffenden Antworten aus:]
    - Tuberculosis [*Tuberkulose*]: 33 (97.06%)
    - Other [*Sonstiges*]: 1 (2.94%)

26. What disease(s) are the hares tested for? (if "hares" to question 22., multiple choice, n=69)
    Please select the answers that apply:
    [*Auf welche Krankheit(en) werden die Hasen untersucht? (falls "Hasen" bei Frage 22 ausgewählt wurde, Mehrfachantwort, n=69)*
    Bitte wählen Sie die zutreffenden Antworten aus:]
    - Tularemia [*Hasenpest/Tularämie*]: 68 (98.55%)
    - Other [*Sonstiges*]: 1 (1.45%)

27. If a disease is suspected in a wild animal, is there a consultation with the cantonal veterinary office before it is sent for investigation of the cause of death/illness? (single choice, n=238)
    Please choose one of the following answers:
    [*Gibt es bei Verdacht auf eine Krankheit bei einem Wildtier eine Absprache mit dem kantonalen Veterinäramt bevor es zur Untersuchung der Todes-/Krankheitsursache eingeschickt wird? – (Einfachantwort, n=238)*
    Bitte wählen Sie eine der folgenden Antworten:]
    - Yes [*Ja*]: 111 (46.64%)
    - No [*Nein*]: 122 (51.26%)
    - No answer [*Keine Antwort*]: 5 (2.10%)

28. What does this consultation look like? (if "yes" to question 27, single choice, n=110)
    Please choose one of the following answers:
    [*Wie sieht diese Absprache aus? (falls "ja" bei Frage 27, Einfachantwort, n=110)*
    Bitte wählen Sie eine der folgenden Antworten:]
    - There is always consultation with the cantonal veterinary office [*Es findet immer eine Absprache mit dem kantonalen Veterinäramt statt*]: 28 (25.45%)
    - Only in case of suspicion of a notifiable disease or in case of the possibility of a transmission to domestic animals a consultation with the cantonal veterinary office takes place [*Nur bei Verdacht auf eine meldepflichtige Krankheit oder bei der Möglichkeit einer Übertragung auf Haustiere findet eine Absprache mit dem kantonalen Veterinäramt statt*]: 69 (62.73%)
    - Other [*Sonstiges*]: 13 (11.82%)

29. Please explain this in more detail: (if "no" to question 27, single choice, n=119)
    Please choose one of the following answers
    [*Erläutern Sie dies bitte genauer: (falls "nein" bei Frage 27, Einfachantwort, n=119)*
    Bitte wählen Sie eine der folgenden Antworten:]
    - The cantonal veterinary office is not involved in the decision whether to send an animal for investigation of the cause of death/disease [*Bei der Entscheidung, ob ein Tier zur Untersuchung der Todes-/Krankheitsursache eingeschickt wird, wird das kantonale Veterinäramt nicht involviert*]: 51 (42.86%)
    - There is no consultation in advance, but the cantonal veterinary office is informed of all submissions [*Es gibt keine Absprache vorab, aber das kantonale Veterinäramt wird über sämtliche Einsendungen informiert*]: 45 (37.82%)
    - There is no consultation in advance, but the cantonal veterinary office is always informed of any suspicion of a notifiable disease [*Es gibt keine Absprache vorab, aber das kantonale Veterinäramt wird immer über den Verdacht einer meldepflichtigen Krankheit informiert*]: 11 (9.24%)
    - Other [*Sonstiges*]: 12 (10.08%)

30. Please indicate the frequency with which you send whole bodies or organs of animals from the following groups to a laboratory/institute for examination of the cause of death/disease (n=238):
1 = never; 2 = 1-5 per year; 3 = 6-10 per year; 4 = 11-20 per year; 5 = >20 per year.
[*Geben Sie bitte an, mit welcher Häufigkeit Sie ganze Körper oder Organe von Tieren aus den folgenden Gruppen an ein Labor/Institut zur Abklärung der Todes-/Krankheitsursache einschicken (n=238):*
1 = nie; 2 = 1-5 pro Jahr; 3 = 6-10 pro Jahr; 4 = 11-20 pro Jahr; 5 = >20 pro Jahr]

	**Ruminants (%)**	**Wild boar (%)**	**Hunted carnivores (%)**	**Raccoon/ raccoon dog (%)**	**Protected carnivores (%)**	**Lagomorphs (%)**	**Protected rodents (%)**	**Hunted rodents (%)**	**Small rodents (%)**	**Eulipotyphla (%)**	**Bats (%)**	**Raptors/owls (%)**	**Waterfowl (%)**	**Songbirds (%)**	**Reptiles (%)**	**Amphibians (%)**
**1**	96 (40.34)	85 (35.71)	83 (34.87)	167 (70.17)	107 (44.96)	106 (45.54)	124 (52.10)	169 (71.01)	171 (71.85)	172 (72.72)	167 (70.17)	126 (52.94)	139 (58.40)	147 (61.76)	157 (65.97)	170 (71.43))
**2**	97 (40.76)	73 (30.67)	108 (45.38)	11 (4.62)	76 (31.93)	90 (37.82)	60 (25.21)	8 (3.36)	8 (3.36)	8 (3.36)	13 (5.46)	56 (23.53)	44 (18.49)	29 (12.18)	12 (5.04)	8 (3.36)
**3**	7 (2.94)	12 (5.04)	11 (4.62)	0	3 (1.26)	5 (2.10)	4 (1.68)	1 (0.42)	1 (0.42)	0	0	0	5 (2.10)	3 (1.26)	1 (0.42)	1 (0.42)
**4**	1 (0.42)	8 (3.36)	2 (0.84)	0	0	0	0	0	0	0	0	0	0	0	0	0
**5**	4 (1.68)	23 (9.66)	0	0	1 (0.42)	0	0	0	0	0	0	0	0	0	0	0
**NA**	33 (13.87)	37 (15.55)	34 (14.29)	60 (25.21)	51 (21.43)	37 (15.55)	50 (21.01)	60 (25.21)	58 (24.37)	58 (24.37)	58 (24.37)	56 (23.53)	50 (21.01)	59 (24.79)	58 (24.37)	59 (24.79)

31. Have you already submitted carcasses with **unknown cause of death** (no evidence of accident) and **no recognizable signs of disease** for clarification of the cause of death/illness (regardless of the animal species)? (single choice, n=238)
Please choose one of the following answers
[*Haben Sie bereits Fallwild/Totfunde mit unbekannter Todesursache (keine Hinweise auf Unfall) und keinen erkennbaren Krankheitsanzeichen zur Abklärung der Todes-/Krankheitsursache eingeschickt (unabhängig von der Tierart)? (Einfachantwort, n=238)*
Bitte wählen Sie eine der folgenden Antworten]
   - Yes [*Ja*]: 131 (55.04%)
   - No [*Nein*]: 105 (44.12%)
   - No answer [*Keine Antwort*]*:* 2 (0.84%)

32. If, within a month, there is an increased number of animals found dead with **unknown cause of death** (no indications of accident) and **no recognizable signs of disease** occur in any of the following groups of game in your supervisory area/district, at what number of deaths do you send in animals for clarification of the cause of death/disease (or would you send in if this has never happened before)? (n=238)
[*Wenn innerhalb eines Monats vermehrt Totfunde/Fallwild mit* ***unbekannter Todesursache*** *(keine Hinweise auf Unfall) und* ***keinen erkennbaren Krankheitsanzeichen*** *bei einer der folgenden Wildgruppen in Ihrem Aufsichtsgebiet/Revier auftreten, ab welcher Anzahl von Todesfällen schicken Sie Tiere zur Abklärung der Todes-/Krankheitsursache ein (bzw. würden Sie einschicken, wenn dies noch nie erfolgt ist)? (n=238)*]

	Ruminants (%)	Wild boar (%)	Hunted carnivores (%)	Raccoon/ raccoon dog (%)	Protected carnivores (%)	Lagomorphs (%)	Protected rodents (%)	Hunted rodents (%)	Small rodents (%)	Eulipotyphla (%)	Bats (%)	Raptors/owls (%)	Waterfowl (%)	Songbirds (%)	Reptiles (%)	Amphibians (%)
**none**	1 (0.42)	13 (5.46)	3 (1.26)	11 (4.62)	4 (1.68)	3 (1.26)	7 (2.94)	23 (9.66)	17 (7.14)	16 (6.72)	19 (7.98)	6 (2.52)	9 (3.78)	10 (4.20)	22 (9.24)	23 (9.66)
**1**	32 (13.45)	87 (36.55)	22 (9.24)	70 (29.41)	157 (65.97)	65 (27.31)	67 (28.15)	19 (7.98)	9 (3.78)	10 (4.20)	12 (5.04)	53 (22.27)	23 (9.66)	19 (7.98)	8 (3.36)	7 (2.94)
**2-3**	122 (51.26)	61 (25.63)	105 (44.12)	53 (22.27)	12 (5.04)	85 (35.71)	71 (29.83)	60 (25.21)	26 (10.92)	58 (24.37)	51 (21.43)	80 (33.61)	78 (32.77)	64 (26.89)	57 (23.95)	36 (15.13)
**4-5**	18 (7.56)	6 (2.52)	37 (15.55)	7 (2.94)	1 (0.42)	18 (7.56)	8 (3.36)	17 (7.14)	42 (17.65)	29 (12.19)	29 (12.18)	15 (6.30)	32 (13.45)	38 (15.97)	21 (8.82)	28 (11.76)
**6-10**	7 (2.94)	2 (0.84)	9 (2.94)	3 (1.26)	0	3 (1.26)	2 (0.84)	3 (1.26)	19 (7.98)	9 (3.78)	10 (4.20)	3 (1.26)	6 (2.52))	8 (3.36)	6 (2.52)	12 (5.04)
**>10**	1 (0.42)	1 (0.42)	2 (1.26)	0	0	1 (0.42)	2 (0.84)	2 (0.84)	11 (4.62)	4 (1.68)	5 (2.10)	0	2 (0.84)	3 (1.26)	4 (1.68)	11 (4.62)
**NA**	37 (15.55)	53 (22.27)	40 (16.81)	77 (32.35)	50 (21.01)	43 (18.07)	64 (26.89)	96 (40.34)	91 (38.24)	93 (39.08)	90 (37.82)	65 (27.31)	69 (28.99)	78 (32.77)	99 (41.60)	99 (41.60)
**After agreement**	14 (5.88)	10 (4.20)	12 (5.04)	9 (3.78)	8 (3.36)	10 (4.20)	9 (3.78)	11 (4.62)	10 (4.20)	9 (3.78)	10 (4.20)	11 (4.62)	10 (4.20)	8 (3.36)	8 (3.36)	8 (3.36)
**other**	6 (2.52)	5 (2.10)	8 (3.36)	8 (3.36)	6 (2.52)	10 (4.20)	8 (3.36)	7 (2.94)	13 (5.46)	10 (4.20)	12 (5.04)	5 (2.10)	9 (3.78)	10 (4.20)	13 (5.46)	14 (5.88)

33. Have you already sent in animals found dead or culled **with recognizable signs of disease** (e.g. mange, distemper) for clarification of the cause of death/disease? (single choice, n=238)
Please choose one of the following answers
[*Haben Sie bereits Fallwild/Totfunde bzw. Hegeabschüsse MIT erkennbaren Krankheitsanzeichen (z.B. Räude, Staupe) zur Abklärung der Todes-/Krankheitsursache eingeschickt? (Einfachantwort, n=238) Bitte wählen Sie eine der folgenden Antworten*]
   - Yes [*Ja*]*:* 134 (56.30%)
   - No [*Nein*]*:* 102 (42.86%)
   - No answer [*Keine Antwort*]*:* 2 (0.84%)

34. For which reason? (if "yes" to question 33, multiple choice, n=134)
[*Aus welchem Grund? (falls "ja" bei Frage 33, Mehrfachantwort, n=134)*]
   - To confirm the suspected diagnosis [*Zur Bestätigung der Verdachtsdiagnose*]*:* 123 (91.79%)
   - Own interest [*Eigenes Interesse]:* 35 (26.12%)
   - Requirement of authority [*Vorgabe der Behörde*]*:* 55 (41.04%)
   - Request from laboratory/institute [*Wunsch von Labor/Institut*]*:* 24 (17.91%)
   - Other [*Sonstiges*]*:* 0

35. For which reason? (if "no" to question 33, multiple choice, n=102)
[*Aus welchem Grund? (falls "nein" bei Frage 33, Mehrfachantwort, n=102)*]
   - I can make the diagnosis myself [*Ich kann die Diagnose selber stellen*]*:* 66 (64.71%)
   - Requirement of authority [*Vorgabe der Behörde*]*:* 13 (12.75%)
   - Request from laboratory/institute [*Wunsch von Labor/Institut*]*:* 3 (2.94%)
   - Other [*Sonstiges*]*:* 0

36. If, within one month, there is an increase in the number of animals found dead (or culled) **with recognizable signs of disease** (e.g. mange, distemper) which occur in any of the following groups of wildlife in your supervisory area/district, at what number of deaths do you send in animals for clarification of the cause of death/disease (or would you send in if this has never happened), if it is likely to **be a disease that has NOT occurred in your area before** (as far as you know)? (n=238)

[*Wenn innerhalb eines Monats vermehrt Totfunde/Fallwild (bzw. Hegeabschüsse) mit* ***erkennbaren Krankheitsanzeichen*** *(z.B. Räude, Staupe) bei einer der folgenden Wildgruppen in Ihrem Aufsichtsgebiet/Revier auftreten, ab welcher Anzahl von Todesfällen schicken Sie Tiere zur Abklärung*

der Todes-/Krankheitsursache ein (oder würden Sie einschicken, wenn dies noch nie erfolgt ist),wenn es sich vermutlich um eine ***Krankheit*** *handelt,* ***die in Ihrem Gebiet (soviel Sie wissen) bisher NICHT vorgekommen ist?****) (n=238)*]

	**Ruminants (%)**	**Wild boar (%)**	**Hunted carnivores (%)**	**Raccoon/ raccoon dog (%)**	**Protected carnivores (%)**	**Lagomorphs (%)**	**Protected rodents (%)**	**Hunted rodents (%)**	**Small rodents (%)**	**Eulipotyphla (%)**	**Bats (%)**	**Raptors/owls (%)**	**Waterfowl (%)**	**Songbirds (%)**	**Reptiles (%)**	**Amphibians (%)**
**none**	3 (1.26)	12 (5.04)	5 (2.10)	13 (5.46)	7 (2.94)	6 (2.52)	7 (2.94)	24 (10.08)	18 (7.56)	17 (7.14)	17 (7.14)	8 (3.36)	9 (3.78)	9 (3.87)	20 (8.40)	20 (8.40)
**1**	81 (34.03)	99 (41.60)	69 (28.99)	87 (36.55)	139 (58.40)	90 (37.82)	90 (37.82)	48 (20.17)	30 (12.61)	35 (14.71)	43 (18.07)	76 (31.93)	52 (21.85)	47 (19.75)	37 (15.55)	35 (14.71)
**2-3**	71 (29.83)	45 (18.91)	73 (30.67)	34 (14.29)	9 (3.78)	45 (18.91)	37 (15.55)	33 (13.87)	29 (12.81)	46 (19.33)	34 (14.29)	53 (22.27)	55 (23.11)	44 (18.49)	32 (13.45)	24 (10.08)
**4-5**	15 (6.30)	5 (2.10)	25 (10.50)	4 (1.68)	1 (0.42)	16 (6.72)	11 (4.62)	13 (5.46)	28 (11.76)	16 (6.72)	21 (8.82)	6 (2.52)	12 (5.04)	16 (6.72)	17 (7.14)	19 (7.98)
**6-10**	0	0	3 (1.26)	2 (0.84)	0	3 (1.26)	3 (1.26)	2 (0.84)	11 (4.62)	8 (3.36)	6 (2.52)	1 (0.42)	5 (2.10)	9 (3.78)	5 (2.10)	7 (2.94)
**>10**	2 (0.84)	2 (0.84)	1 (0.42)	2 (0.84)	1 (0.42)	0	1 (0.42)	3 (1.26)	7 (2.94)	3 (1.26)	4 (1.68)	2 (0.84)	4 (1.68)	5 (2.10)	3 (1.26)	8 (3.36)
**NA**	43 (18.07)	59 (24.79)	41 (17.23)	81 (34.03)	66 (27.73)	61 (25.63)	73 (39.67)	98 (41.18)	93 (39.08)	93 (39.08)	91 (38.24)	76 (31.93)	83 (34.87)	87 (36.55)	102 (42.86)	101 (42.44)
**After agreement**	14 (5.88)	9 (3.78)	13 (5.46)	9 (3.78)	9 (3.78)	10 (4.20)	8 (3.36)	10 (4.20)	10 (4.20)	10 (4.20)	10 (4.20)	10 (4.20)	11 (4.62)	10 (4.20)	9 (3.78)	8 (3.36)
**other**	9 (3.78)	7 (2.94)	8 (3.36)	6 (2.52)	6 (2.52)	7 (2.94)	8 (3.36)	7 (2.94)	12 (5.04)	10 (4.20)	12 (5.04)	6 (2.52)	7 (2.94)	11 (4.62)	13 (5.46)	16 (6.72)

37. If, within one month, there is an increase in the number of animals found dead (or culled) **with recognizable signs of disease** (e.g. mange, distemper) which occurs in any of the following groups of wildlife in your supervisory area/district, at what number of deaths do you send in animals for investigation of cause of death/disease (or would you send in if this has never happened), if it is likely to be a disease that **has already occurred in your area**? (n=238)

[*Wenn innerhalb eines Monats vermehrt Totfunde/Fallwild (bzw. Hegeabschüsse)* ***mit erkennbaren Krankheitsanzeichen*** *(z.B. Räude, Staupe) bei einer der folgenden Wildgruppen in Ihrem Aufsichtsgebiet/Revier auftreten, ab welcher Anzahl von Todesfällen schicken Sie Tiere zur Untersuchung der Todes-/Krankheitsursache ein (oder würden Sie einschicken, wenn dies noch nie erfolgt ist), wenn es sich vermutlich um eine* ***Krankheit*** *handelt,* ***die in Ihrem Gebiet bereits vorgekommen ist?****) (n=238)*]

	**Ruminants (%)**	**Wild boar (%)**	**Hunted carnivores (%)**	**Raccoon/ raccoon dog (%)**	**Protected carnivores (%)**	**Lagomorphs (%)**	**Protected rodents (%)**	**Hunted rodents (%)**	**Small rodents (%)**	**Eulipotyphla (%)**	**Bats (%)**	**Raptors/owls (%)**	**Waterfowl (%)**	**Songbirds (%)**	**Reptiles (%)**	**Amphibians (%)**
**none**	10 (4.20)	18 (7.56)	13 (5.46)	16 (6.72)	12 (5.04)	14 (5.88)	12 (5.04)	33 (13.87)	24 (10.08)	24 (10.08)	24 (10.08)	15 (6.30)	16 (6.72)	18 (7.56)	27 (11.34)	27 (11.34)
**1**	38 (15.97)	65 (27.31)	28 (11.76)	55 (23.11)	111 (46.64)	46 (19.33)	54 (22.69)	21 (8.82)	16 (6.72)	18 (7.56)	19 (7.98)	43 (18.07)	30 (12.61)	26 (10.92)	13 (5.46)	13 (5.46)
**2-3**	65 (27.31)	40 (16.81)	52 (21.85)	33 (13.87)	16 (6.72)	47 (19.75)	45 (18.91)	34 (14.29)	25 (10.50)	36 (15.13)	32 (13.45)	47 (19.75)	39 (16.39)	39 (16.39)	33 (13.87)	28 (11.46)
**4-5**	26 (10.92)	17 (7.14)	34 (14.29)	18 (7.56)	5 (2.10)	22 (9.24)	13 (5.46)	16 (6.72)	26 (10.92)	24 (10.08)	20 (8.40)	16 (6.72)	27 (11.34)	24 (10.08)	22 (9.24)	20 (8.40)
**6-10**	8 (3.36)	6 (2.52)	16 (6.72)	6 (2.52)	4 (1.68)	8 (3.36)	9 (3.78)	8 (3.36)	20 (8.40)	14 (5.88)	13 (5.46)	7 (2.94)	6 (2.52)	9 (3.78)	9 (3.78)	10 (4.20)
**>10**	5 (2.10)	3 (1.26)	8 (3.36)	4 (1.68)	2 (0.84)	6 (2.52)	3 (1.26)	5 (2.10)	9 (3.78)	4 (1.68)	7 (2.94)	4 (1.68)	7 (2.94)	8 (3.36)	6 (9.24)	11 (4.62)
**NA**	62 (26.05)	73 (30.67)	63 (26.47)	90 (37.82)	75 (31.51)	76 (31.93)	85 (35.71)	105 (44.12)	98 (41.18)	100 (42.44)	101 (42.44)	89 (37.39)	95 (39.92)	95 (39.92)	111 (46.64)	113 (47.48)
**After agreement**	13 (5.46)	8 (3.36)	11 (4.62)	9 (3.78)	8 (3.36)	10 (4.20)	7 (2.94)	9 (3.78)	10 (4.20)	10 (4.20)	11 (4.62)	9 (3.78)	10 (4.20)	10 (4.20)	7 (2.94)	6 (2.52)
**other**	11 (4.62)	8 (3.36)	13 (5.46)	7 (2,^.94)	5 (2.10)	9 (3.78)	10 (4.20)	7 (2.94)	10 (4.20)	8 (3.36)	11 (4.62)	8 (3.36)	8 (3.36)	9 (3.78)	10 (4.20)	10 (4.20)

38. On average, how many carcasses with clear evidence of an accident do you send in for necropsy each year? Comment when an answer is selected (multiple choice, n=238)
[*Wieviel Fallwild/Totfunde mit eindeutigen Hinweisen auf einen Unfall schicken Sie durchschnittlich pro Jahr zur Sektion ein? Kommentieren wenn eine Antwort gewählt wird (Mehrfachantwort, n=238)*]
   - None (unless specifically requested by FIWI, the cantonal veterinary office or another institution) [*Keine (ausser es wird speziell vom FIWI, dem kantonalen Veterinäramt bzw. einer anderen Institution gewünscht)*]*:* 174 (73.11%)
   - Approximate number of animals [*Anzahl Tiere ungefähr*]
     - 0: 2 (0.84%)
     - 1-2: 16 (6.72%)
     - 3-4: 12 (5.04)
     - 5-10: 16 (6.72%)
     - >10: 4 (1.68%)

39. Which species mainly? (if "approximate number of animals" was given in question 38., n=48)
[*Welche Tierarten hauptsächlich? (falls "Anzahl Tiere ungefähr" gewählt wurde bei Frage 38, n=48)*]
   - Ruminants [*Wiederkäuer*]*:* 21 (43.75%)
   - Wild boars [*Wildschweine*]*:* 10 (20.83%)
   - Hunted carnivores [*Jagdbare Raubtiere*]*:* 30 (62.50%)
   - Protected carnivores [*Geschützte Raubtiere*]*:* 17 (35.42%)
   - Lagomorphs [*Hasenartige*]*:* 14 (29.17%)
   - Protected rodents [*Geschützte Raubtiere*]: 11 (22.95%)
   - Birds of prey [*Greifvögel*]*:* 12 (25%)
   - Waterfowl [*Wasservögel*]*:* 6 (12.50%)
   - Songbirds [*Singvögel*]*:* 2 (4.17%)
   - Diagnostic of predation [*Rissdiagnostik*]*:* 1 (2.08%)

40. Has the number of cases you send in for examination of the cause of death/illness changed since you started working in your current supervisory area/district/institution? (single choice, n=238)
Please select one of the following answers:
[*Hat sich die Anzahl der Fälle, die Sie zur Abklärung der Todes-/Krankheitsursache einschicken, seit Sie im jetzigen Aufsichtsgebiet/Revier bzw. Institution tätig sind geändert? (Einfachantwort, n=238)*
Bitte wählen Sie eine der folgenden Antworten:]
   - No, the number of submissions has stayed about the same [*Nein, die Anzahl der Einsendungen ist ungefähr gleich geblieben*]: 184 (77.31%)
   - Yes, it has decreased [*Ja, sie hat abgenommen*]*:* 19 (7.98%)
   - Yes, it has increased [*Ja, sie hat zugenommen*]*:* 20 (8.40%)

41. What reasons do you suspect for this decrease? (if "yes, it has decreased" was checked in question 40., multiple choice, n=19)
Please explain your answers in more detail in each case or give an example.
Comment if an answer is selected
[*Welche Gründe vermuten Sie für diese Abnahme? (falls "ja, sie hat abgenommen" gewählt wurde bei Frage 40, Mehrfachantwort, n=19)*
Bitte erläutern Sie Ihre Antworten jeweils genauer oder geben Sie ein Beispiel.
Kommentieren wenn eine Antwort gewählt wird)]
   - Fewer dead/diseased animals (decrease in dead animals) [*Weniger tote/erkrankte Tiere (Abnahme der Totfunde)*]*:* 10 (52.63%)
   - Own interest has changed [*Eigenes Interesse hat sich geändert*]*:* 2 (10.53%)
   - Change in legal basis [*Änderung der gesetzlichen Grundlage*]*:* 1 (5.26%)
   - Other instructions from hunting management [*Andere Anweisungen der Jagdverwaltung*]*:* 8 (42.11%)
   - Other [*Sonstiges*]*:* 2 (10.53%)

42. What reasons do you suspect for this increase? (if "yes, it has increased" was checked in question 40., multiple choice, n=20)
Please explain your answers in more detail in each case or give an example.
Comment if an answer is selected
[*Welche Gründe vermuten Sie für diese Abnahme? (falls "ja, sie hat abgenommen" gewählt wurde bei Frage 40, Mehrfachantwort, n=20)*
Bitte erläutern Sie Ihre Antworten jeweils genauer oder geben Sie ein Beispiel.
Kommentieren wenn eine Antwort gewählt wird]
    - More dead/diseased animals (increase in dead animals) [*Mehr tote/erkrankte Tiere (Zunahme der Totfunde)*]*:* 9 (45%)
    - Increase in the population of protected animal species that must be examined at FIWI according to the FOEN (bear, wolf, lynx, wildcat) [*Zunahme der Population geschützter Tierarten, die gemäss BAFU am FIWI untersucht werden müssen (Bär, Wolf, Luchs, Wildkatze)*]*:* 11 (55%)
    - Higher own awareness of diseases through information/education (e.g. Avian influenza, tuberculosis, African swine fever, distemper) [*Höheres eigenes Bewusstsein für Krankheiten durch Informationen/Weiterbildungen (z.B. Vogelgrippe, Tuberkulose, Afrikanische Schweinepest, Staupe)*]*:* 13 (65%)
    - Own interest has changed [*Eigenes Interesse hat sich geändert*]*:* 3 (15%)
    - Change in legal basis [*Änderung der gesetzlichen Grundlage*]*:* 0
    - Other instructions from hunting management [*Andere Anweisungen der Jagdverwaltung*]*:* 5 (25%)
    - Other [*Sonstiges*]*:* 2 (10%)

43. In general, how do you assess the current support by the responsible persons in your canton and other institutions regarding the question of whether or not to send in an animal for examination of the cause of death/illness? (single choice, n=238) Please choose one of the following answers:
[*Wie beurteilen Sie allgemein die gegenwärtige Unterstützung durch die verantwortlichen Personen in Ihrem Kanton und andere Institutionen bei der Frage, ob Sie ein Tier zur Abklärung der Todes-/Krankheitsursache einschicken sollen oder nicht? (Einfachantwort, n=238)*
Bitte wählen Sie eine der folgenden Antworten:]
    - Very good [*Sehr gut*]*:* 132 (55.46%)
    - Good [*Gut*]*:* 66 (27.73%)
    - Sufficient [*Ausreichend*]*:* 27 (11.34%)
    - Poor [*Schlecht*]*:* 3 (1.26%)
    - Very bad [*Sehr schlecht*]*:* 1 (0.42%)
    - No answer [*Keine Antwort*]*:* 9 (3.78%)

44. From which institution would you like more assistance in determining whether or not to send an animal in for examination of the cause of death/disease? (multiple choice, n=238)
Please select the answers that apply:
[*Von welcher Institution wünschen Sie sich mehr Unterstützung bei der Frage, ob Sie ein Tier zur Abklärung der Todes-/Krankheitsursache einschicken sollen oder nicht?(Mehrfachantwort, n=238)*
Bitte wählen Sie die zutreffenden Antworten aus:]
    - None, everything is fine for me [*Keiner, für mich stimmt alles so*]*:* 182 (76.47%)
    - Cantonal hunting administration [*Kantonale Jagdverwaltung*]*:* 28 (11.76%)
    - Cantonal veterinary office [*Kantonales Veterinäramt*]*:* 30 (12.61%)
    - Private veterinarian [*Privattierarzt/in*]*:* 1 (0.42%)
    - Federal Food Safety and Veterinary Office (FSVO) [*Bundesamt für Lebensmittelsicherheit und Veterinärwesen (BLV)*]*:*4 (1.68%)
    - Federal Office for the Environment (FOEN) [*Bundesamt für Umwelt (BAFU)*]*:* 10 (4.20%)
    - Institute for Fish and Wildlife Health (FIWI) [*Institut für Fisch- und Wildtiergesundheit (FIWI)*]*:* 20 (8.40%)

- Cantonal laboratory [*Kantonales Labor*]*:* 1 (0.42%)
- Other [*Sonstiges*]*:* 1 (0.42%)

45. Which cantonal laboratory? (n=1)
    [*Welches kantonale Labor? (n=1)*]
    - Service de la consommation et des affaires vétérinaires, Neuchatel

46. Would you consider it useful to have a nationwide electronic reporting system for wild animals found dead/culled, in which cases not sent in could be recorded more precisely than in the hunting statistics (possibly a system linked to the hunting statistics)? (single choice, n=238)
    Please give reasons for your answer.
    Please choose one of the following answers:
    [*Würden Sie ein schweizweites elektronisches Meldesystem für tot aufgefundene/krank erlegte Wildtiere, in dem nicht eingesandte Fälle genauer erfasst werden könnten als in der Jagdstatistik (evtl. mit der Jagdstatistik verknüpftes System) als sinnvoll erachten? (Einfachantwort, n=238)*
    Bitte begründen Sie Ihre Antwort.
    Bitte wählen Sie eine der folgenden Antworten:]
    - Yes [*Ja*]*:* 103 (43.28%)
    - No [*Nein*]*:* 126 (52.94%)
    - No answer [*Keine Antwort*]*:* 9 (3.78%)

47. Do you have any questions or comments about this questionnaire or the FIWI?
    [*Haben Sie Fragen oder Bemerkungen zu diesem Fragebogen oder dem FIWI?*]
