# Supplementary material for: Analysis of a European general wildlife health surveillance program: Chances, challenges and recommendations
Source: PLoS One. 2024 May 21;19(5):e0301438. doi: 10.1371/journal.pone.0301438 (PMC11108157; doi:10.1371/journal.pone.0301438)
Supplement: S3 Appendix — (PDF) [file pone.0301438.s003.pdf]

Questions on the needs assessment of hunting administrations

Initial situation/objective

The present questionnaire is sent to the cantonal hunting administrators in addition to the online questionnaire on wildlife submissions, which is addressed to the submitters of carcasses. It is important to the Federal Office for the Environment as the commissioner that the wildlife health surveillance offered at the diagnostic institute (FIWI) meets both the legal requirements and the needs of the partners involved. This concerns both the need for investigations at the FIWI and for information on current disease outbreaks in Switzerland.

Investigations at the FIWI

1. Do the services offered at the FIWI cover your needs for veterinary examinations of free-ranging wildlife?

    a. If no, in which area do you have additional needs?

2. Which cases do you send to the FIWI for examination? Which ones do you have examined in your canton (e.g. in a cantonal laboratory)?

3. What cantonal capacities would there be for examinations that would otherwise be done at the FIWI?

Information on the current wildlife health situation

4. Do you have the necessary information on the current health situation of wild mammals and birds in Switzerland/your canton available in an appropriate way?

    a. If no, where specifically do you need more information?

    b. What should the information look like (e.g. monthly newsletter, online system)?

    c. Are the quarterly reports from the FIWI sufficient for you?

5. Are the information on the current health situation of wild mammals and birds in Europe available to you in an appropriate way (e.g. Radar Bulletin from the Food Safety and Veterinary Office)?

6. Are you familiar with the Radar Bulletin of the Food Safety and Veterinary Office, which gives information on the animal disease situation abroad?

7. How does the mutual exchange with neighboring cantons or countries take place regarding the health situation of wild mammals and birds?

    a. Is there a need to expand this exchange at the moment?

Online reporting system

8. Do you record animals found dead or being culled digitally in your canton? (Does anything like this exist in your canton?)

    a. Are specific diseases recorded? If yes, which ones?

9. Could you imagine a nationwide online reporting system for animals found dead or diseased, in which cases that are not sent in for postmortem investigation can be recorded more precisely than in the hunting statistics? (The hunting statistics currently only allows the indication of "age, illness, weakness").

10. Should such an online reporting system be linked to the hunting statistics?
