# Supplementary material for: Analysis of a European general wildlife health surveillance program: Chances, challenges and recommendations
Source: PLoS One. 2024 May 21;19(5):e0301438. doi: 10.1371/journal.pone.0301438 (PMC11108157; doi:10.1371/journal.pone.0301438)
Supplement: S4 Appendix — (PDF) [file pone.0301438.s004.pdf]

Questions on the needs assessment of non-cantonal institutions

Initial situation/objectives

It is important to the Federal Office for the Environment as the comissioner that the wildlife health surveillance offered at the diagnostic institute (FIWI) meets both the legal requirements and the needs of the partners involved. This concerns both the need for investigations at the FIWI and for information on current disease outbreaks in Switzerland.

Investigations at the FIWI

1. Do the services offered at FIWI cover your needs for veterinary examinations of free-ranging wildlife?

    a. If no, in which area do you have additional needs?

2. Which cases do you send to the FIWI for examination? Which ones do you have examined in your canton (e.g. in a cantonal laboratory)?

3. What cantonal capacities would there be for examinations that would otherwise be done at the FIWI

Information on the current wildlife health situation

4. Do you have the necessary information on the current health situation of wild mammals and birds in Switzerland/your canton available in an appropriate way?

    a. If no, where specifically do you need more information?

    b. What should the information look like (e.g. monthly newsletter, online system)?

5. Are you familiar with the Radar Bulletin of the Food Safety and Veterinary Office, which gives information on the animal disease situation abroad?

6. Are the information on the current health situation of wild mammals and birds in Europe available to you in an appropriate way (e.g. Radar Bulletin from the Food Safety and Veterinary Office)?

Online reporting system

7. Could you imagine a nationwide online reporting system for free-ranging animals found dead/euthanized or sick?
