# Supplementary material for: Analysis of a European general wildlife health surveillance program: Chances, challenges and recommendations
Source: PLoS One. 2024 May 21;19(5):e0301438. doi: 10.1371/journal.pone.0301438 (PMC11108157; doi:10.1371/journal.pone.0301438)

Criteria for the submission of free-ranging mammals and birds within the general health surveillance

The decision tree applies only to submissions to the **general health surveillance** system of **free-ranging wildlife** (GWHS).

The decisions and criteria do not apply to:

- carcasses where cause of death is suspected to align with targeted surveillance programs (avian influenza, African swine fever, tuberculosis, etc.).
- carcasses of domestic, farm, or wild animals with suspicion of predation as cause of death
- carcasses with suspicion of illegal activities (e.g. poisoning, animal welfare and poaching) as cause of death

In case of uncertainty with regard to the applicable scenario, contacting the diagnostic institute is recommended. In case of suspicion of a reportable animal disease, pre-submission contact with the cantonal veterinary authority is required.

Submissions to support training (e.g., confirmation of a field diagnosis) may be admissible in individual cases.

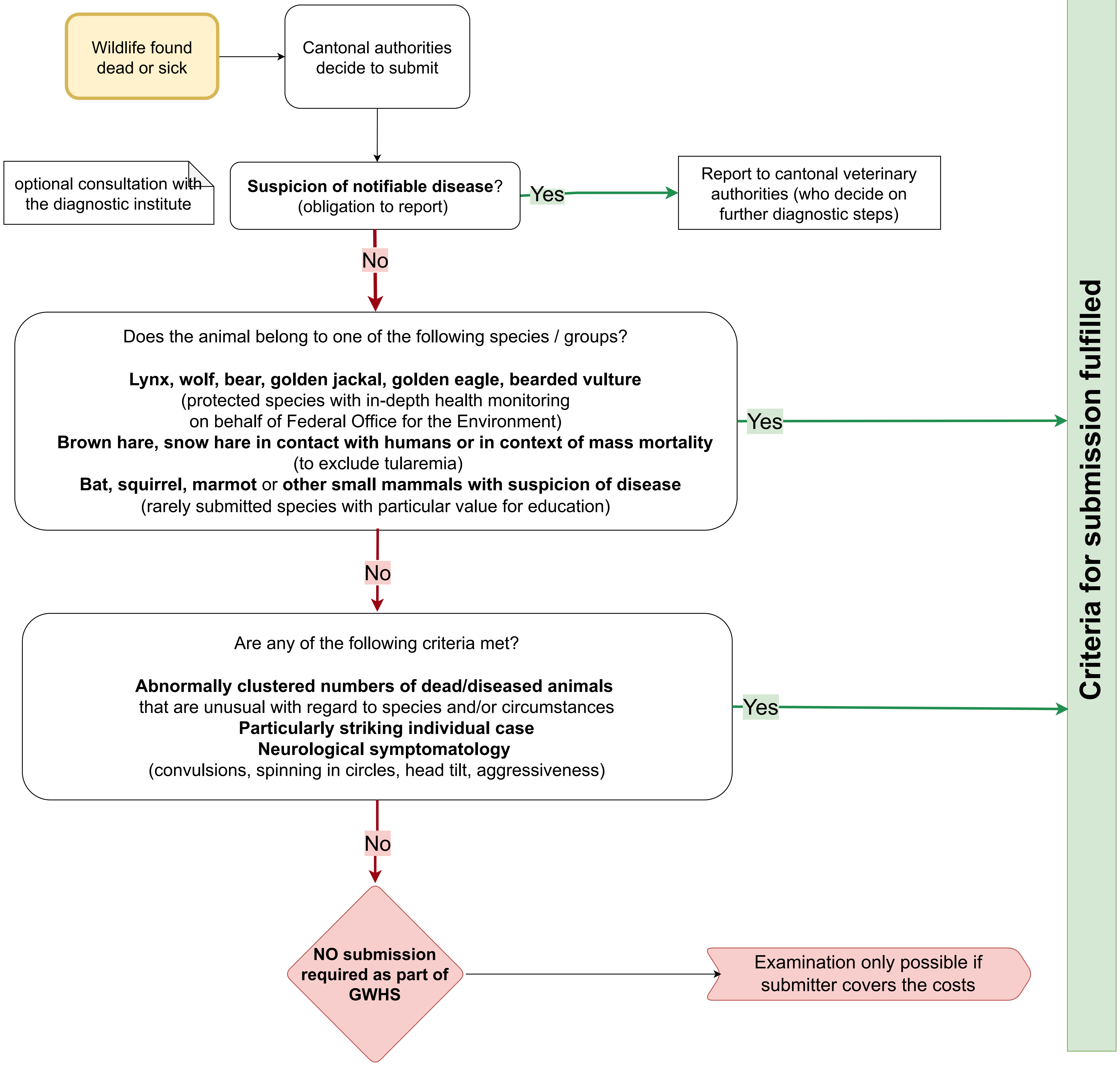

Supplement: S7 Appendix — (PDF) [file pone.0301438.s007.pdf]
